# An LTR Retrotransposon-Derived Gene Displays Lineage-Specific Structural and Putative Species-Specific Functional Variations in Eutherians

**DOI:** 10.3389/fchem.2016.00026

**Published:** 2016-06-23

**Authors:** Masahito Irie, Akihiko Koga, Tomoko Kaneko-Ishino, Fumitoshi Ishino

**Affiliations:** ^1^Department of Nursing, School of Health Sciences, Tokai UniversityIsehara, Japan; ^2^Department of Epigenetics, Medical Research Institute, Tokyo Medical and Dental UniversityTokyo, Japan; ^3^Department of Cellular and Molecular Biology, Primate Research Institute, Kyoto UniversityInuyama, Japan

**Keywords:** LTR retrotransposon, genome evolution, exaptation, primate, brain functions

## Abstract

Amongst the 11 eutherian-specific genes acquired from a sushi-ichi retrotransposon is the CCHC type zinc-finger protein-encoding gene *SIRH11/ZCCHC16*. Its contribution to eutherian brain evolution is implied because of its involvement in cognitive function in mice, possibly via the noradrenergic system. Although, the possibility that *Sirh11/Zcchc16* functions as a non-coding RNA still remains, dN/dS ratios in pairwise comparisons between its orthologs have provided supportive evidence that it acts as a protein. It became a pseudogene in armadillos (Cingulata) and sloths (Pilosa), the only two extant orders of xenarthra, which prompted us to examine the lineage-specific variations of *SIRH11/ZCCHC16* in eutherians. We examined the predicted SIRH11/ZCCHC16 open reading frame (ORF) in 95 eutherian species based on the genomic DNA information in GenBank. A large variation in the SIRH11/ZCCHC16 ORF was detected in several lineages. These include a lack of a CCHC RNA-binding domain in its C-terminus, observed in gibbons (Hylobatidae: Primates) and megabats (Megachiroptera: Chiroptera). A lack of the N-terminal half, on the other hand, was observed in New World monkeys (Platyrrhini: Primates) and species belonging to New World and African Hystricognaths (Caviomorpha and Bathyergidae: Rodents) along with Cetacea and Ruminantia (Cetartiodactyla). Among the hominoids, interestingly, three out of four genera of gibbons have lost normal *SIRH11/ZCCHC16* function by deletion or the lack of the CCHC RNA-binding domain. Our extensive dN/dS analysis suggests that such truncated SIRH11/ZCCHC16 ORFs are functionally diversified even within lineages. Combined, our results show that *SIRH11/ZCCHC16* may contribute to the diversification of eutherians by lineage-specific structural changes after its domestication in the common eutherian ancestor, followed by putative species-specific functional changes that enhanced fitness and occurred as a consequence of complex natural selection events.

## Introduction

Mutation and selection are two principal factors in the Darwinian theory of evolution. The domestication of long terminal repeat (LTR) retrotransposons and retroviruses is a kind of mutation that promotes macroevolution through diversification of genomic function by creating new host genes from exogenous genetic materials (Kaneko-Ishino and Ishino, 2012, 2015; Lavialle et al., 2013; Imakawa et al., 2015). In addition to the investigation of duplication of genes (Ohno, 1970; Kimura, 1983), such acquired genes afford good examples for studying macroevolution and diversification as well as serving as lineage-specific markers in phylogenic analysis. In the human genome, there are approximately 30 LTR retrotransposon-derived genes belonging to two main groups, the sushi-ichi retrotransposon homologs (*SIRH*, also called *MART*, or *SUSHI*) and the paraneoplastic Ma antigen *(PNMA*) family (Voltz et al., 1999; Rosenfeld et al., 2001; Schüller et al., 2005; Campillos et al., 2006; Kaneko-Ishino and Ishino, 2012, 2015; Iwasaki et al., 2013). These genes are derivatives from the original LTR retrotransposons, but each member has a unique DNA sequence. Therefore, each seems to be domesticated in such a manner to have its unique function. Among the *SIRH* genes, *PEG10/SIRH1* (*Paternally expressed 10*) is a therian-specific gene, which is conserved in eutherians and marsupials and plays an essential role in early placenta formation (Ono et al., 2001, 2006; Suzuki et al., 2007). Among all the other eutherian-specific *SIRH* genes, *PEG11/RTL1/SIRH2* (*Paternally expressed 11/Retrotransposon-like 1*) and *SIRH7/LDOC1* (*Leucine zipper, downregulated in cancer 1*) also have been shown to have essential placental functions (Charlier et al., 2001; Edwards et al., 2008; Kagami et al., 2008; Sekita et al., 2008; Naruse et al., 2014), such as maintenance of fetal capillaries and the differentiation/maturation of a variety of placental cells, respectively. All of this evidence provides strong support for the contribution of *SIRH* genes to the evolution of viviparity in mammals via their eutherian-specific functions (Kaneko-Ishino and Ishino, 2012, 2015).

*SIRH11/ZCCHC16* (*Zinc-finger CCHC domain-containing 16*) is an X-linked gene that encodes a CCHC type of zinc-finger protein that exhibits high sequence identity to the LTR retrotransposon Gag protein (Irie et al., 2015). It is expressed in the brain, kidney, testis and ovary in adult mice, and its deletion causes abnormal mouse behaviors related to cognition, including attention, impulsivity and working memory, possibly via the locus coeruleus–noradrenaergic (LC-NA) system (Irie et al., 2015). It is proposed that phasic activation of NA neurons in the LC is linked to cognitive shifts that facilitate dynamic reorganization of target neural networks, thus permitting rapid behavioral adaptation in response to changing environmental imperatives (Berridge and Waterhouse, 2003; Bouret and Sara, 2005). Therefore, we suggest that the acquisition of *SIRH11/ZCCHC16* has played a role in eutherian brain evolution (Irie et al., 2015; Kaneko-Ishino and Ishino, 2015).

The possibility that *Sirh11/Zcchc16* functions as a non-coding RNA has not been completely excluded. The dN/dS ratio is a good indicator of selective pressure acting on a protein-coding gene, calculated as the ratio of the number of nonsynonymous substitutions per nonsynonymous site, in a given period of time, to the number of synonymous substitutions per synonymous site, in the same period. The values < 1 mean that the gene questioned is subjected to purifying selection because the former changes tend to change the protein function while latter changes have no impacts on it. In the case of SIRH11/ZCCHC16, dN/dS ratios in pairwise comparisons of the orthologs between the mouse and seven representative eutherian species other than xenarthran species is approximately 0.35–0.45 (< 1), which suggests that SIRH11/ZCCHC16 has undergone purifying selection after its domestication (exaptation) in the common eutherian ancestor (Irie et al., 2015).

The evolution of mammalian species is associated with several critical geological events and their associated environmental and geographical impacts. The split of the therians from the monotremes occurred 166–186 Mya, followed by the eutherian/marsupial split 160 Mya (Luo et al., 2003; Asher et al., 2004; Madsen, 2009). The domestication of *PEG10* occurred between these two periods (Suzuki et al., 2007) and all the other *SIRH* genes, such as *PEG11/RTL1, SIRH7/LDOC1*, and *SIRH11/ZCCHC16*, were domesticated after the eutherian/marsupial split and before the split of the three major eutherian lineages, boreoeutheria (including euarchontoglires and laurasiatheria), afrotheria and xenarthra 120 Mya that was associated with the division of the supercontinent Pangea (Edwards et al., 2008; Nishihara et al., 2009; Naruse et al., 2014; Irie et al., 2015).

After the extinction of the dinosaurs at the Kreide (Cretaceous)-Paleogene (K-Pg) boundary 65 Mya, an adaptive radiation of mammals independently took place in Eurasia, North and South America, Africa, Australia and Antarctica as well as several isolated islands (Murphy and Eduardo, 2009). The long-term isolation of Australia and South America from other continents as well as the reunion of the two continents, such as Eurasia and Africa, and South and North America, affected the subsequent evolutionary route and history of the eutherians as well as other organisms to a great extent. For example, Xenarthrans evolved and diverged on the isolated South American continent, where carnivorous marsupials and birds had long predominated (Patterson and Pascual, 1972; Murphy and Eduardo, 2009). After the Isthmus of Panama emerged ~3 Mya, the carnivorous marsupials were replaced by an invading carnivorous laurasiatherian species from North America (Patterson and Pascual, 1972). As mentioned, the domestication of all the *SIRH* genes was completed by the time of the emergence of the common eutherian ancestor, after which extensive eutherian diversification occurred in a lineage- and species-specific manner (Kaneko-Ishino and Ishino, 2012, 2015). Therefore, it is of great interest to examine the lineage-specific variations of *SIRH11/ZCCHC16* model gene to explore the extent of its involvement in the eutherian diversification process.

## Results

### Conservation of *SIRH11/ZCCHC16* in eutherian species

**Figure 1:**
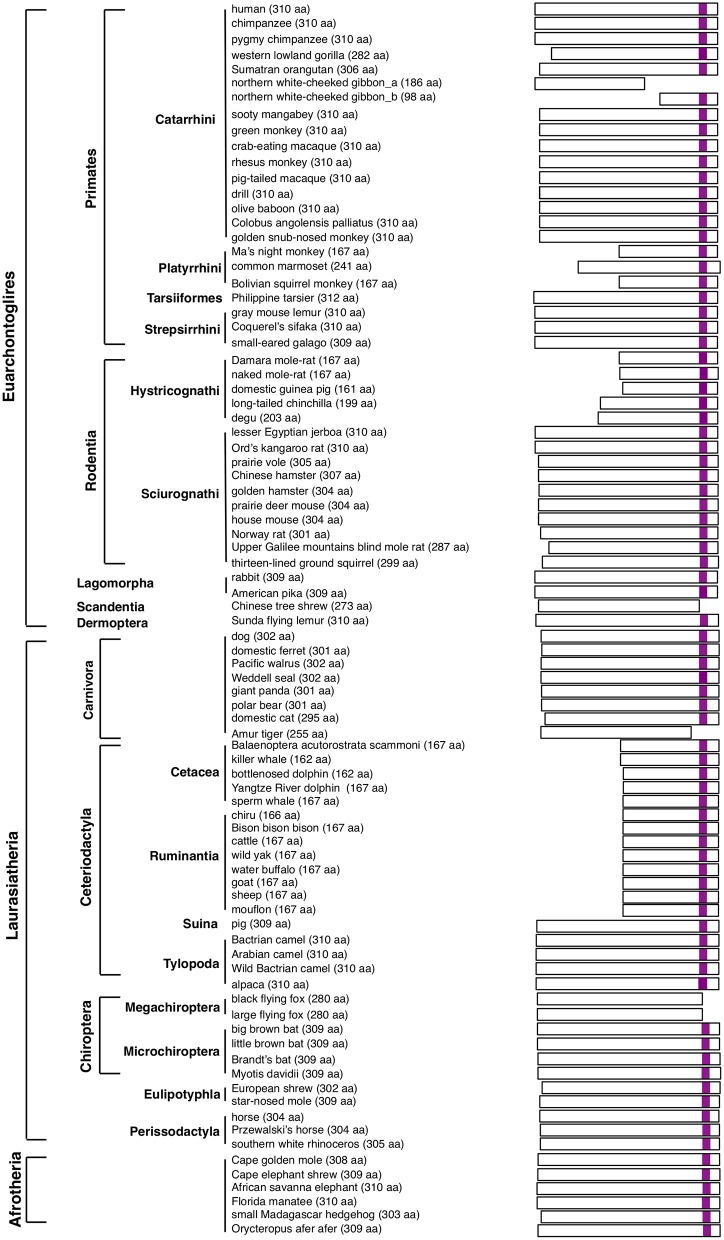
**The scheme represents expected SIRH11 ORF in 83 eutherian mammals**. The purple boxes indicate CCHC RNA-binding domain.

*SIRH11/ZCCHC16* encodes a protein composed of approximately 300–310 amino acids (aa), with a CCHC RNA-binding domain in its C-terminus (Irie et al., 2015). Based on whole genome sequence data of 85 eutherian species from GenBank, including two xenarthran species, the SIRH11/ZCCHC16 ORF in each species was deduced from its own DNA sequence that displayed homology with human *SIRH11/ZCCHC16*. The predicted ORFs in the 83 species, excluding the two xenarthran species with the *pseudoSIRH11/ZCCHC16*, are illustrated in Figure 1 (see also Figure S1: SIRH11/ZCCHC16 aa sequence). Although, there might be some sequence errors in the genomic information, we used it to perform an initial investigation. Ideally, DNA sequences from multiple individuals should be analyzed in every species in this type of investigation. Instead, in this work, we focused on lineage-specific cases and confirmed the mutations using genomic DNA from the same as well as additional species in some of the primate lineages. As a result, a total of 95 eutherian species were analyzed in this study. Afrotheria is the most closely related eutherian group to the xenarthrans and their SIRH11/ZCCHC16 ORFs are highly conserved, as previously reported (Figure 1, the lowest of the six rows).

### Mutations leading to the loss of a CCHC RNA-binding domain in the boreotherians

Nonsense mutations leading to loss of a CCHC RNA-binding domain were observed in five boreotherian [two euarchontoglires and three laurasiatherian species, including the white-cheeked gibbon (Primates), Chinese tree shrew (Scandentia), Amur tiger (Carnivora), and two flying fox species (Chiroptera)] (Figure 1, Figure S1). It is possible that these mutations may be due to sequence errors, however, it is worth considering other possibilities, including the cases in which the mutations are lineage-specific, such as the gibbons and two megabat species (Megachiroptera). Our analyses suggest that this type of mutation changes *SIRH11/ZCCHC16* function at least in these lineages. Interestingly, in one gibbon species, the truncated *SIRH11/ZCCHC16* is suggested to have become pseudogenized and it was lost from two other gibbon species by profound structural changes.

**Figure 2:**
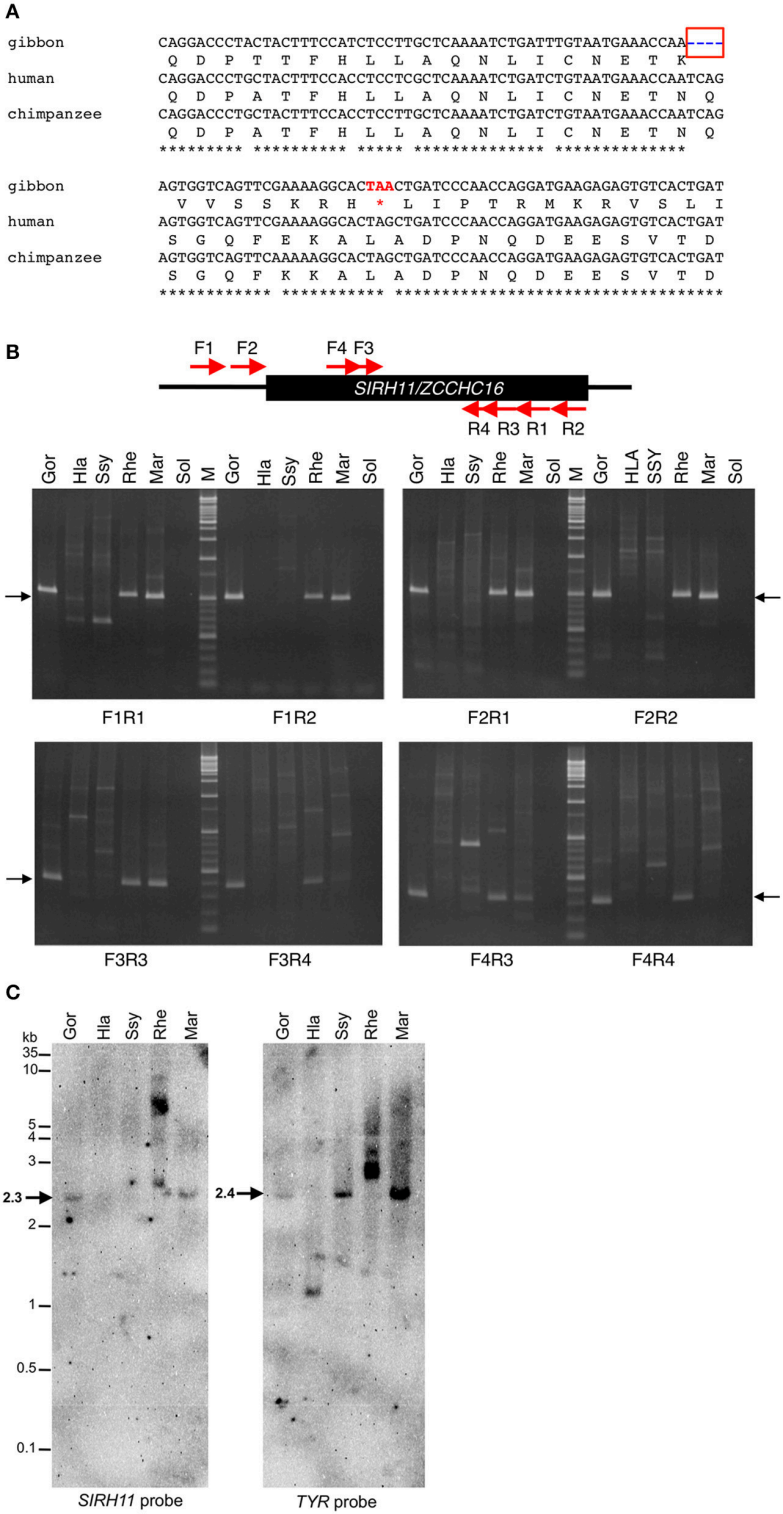

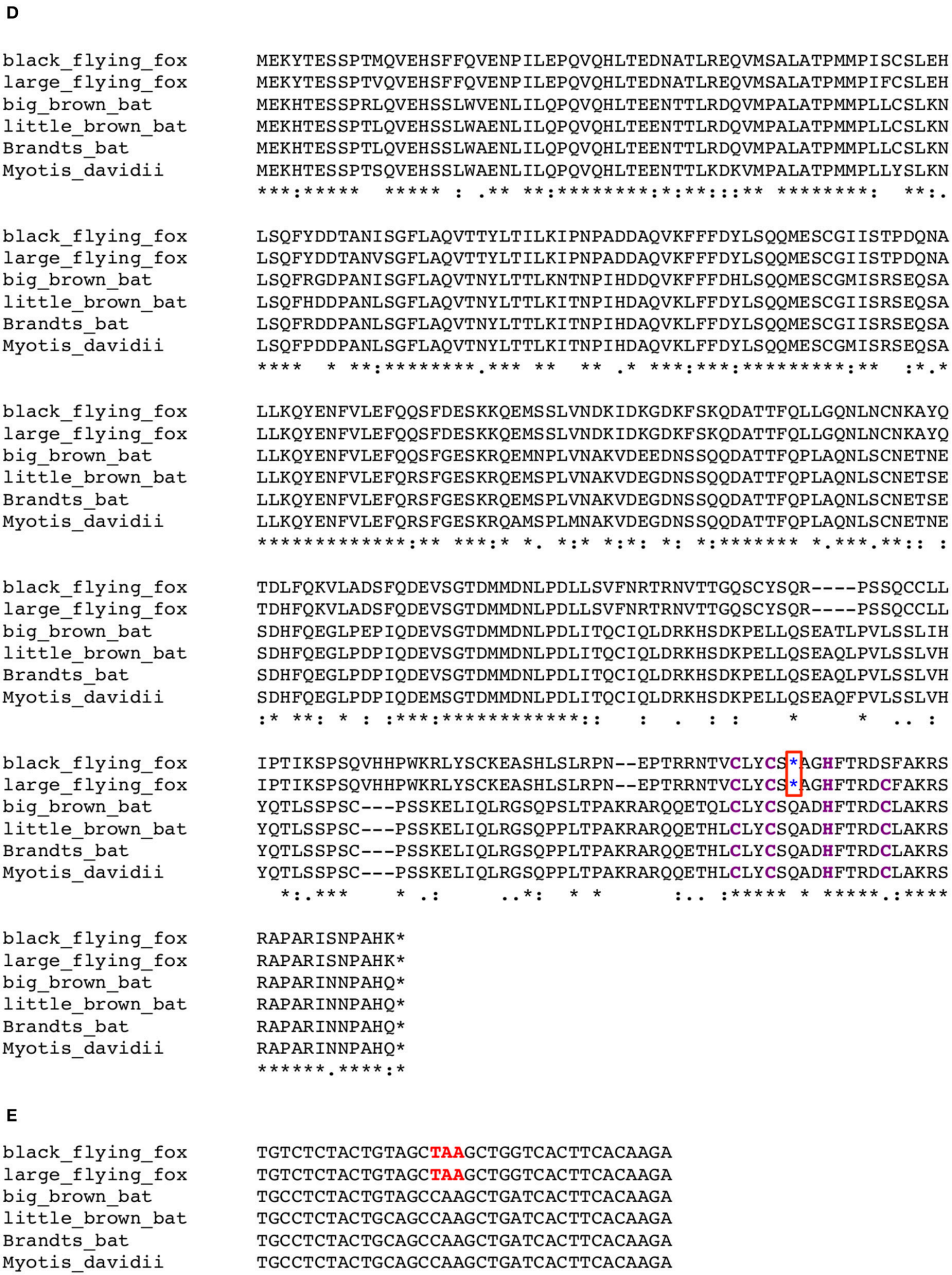
**(A)** Nonsense mutation in gibbon *SIRH11/ZCCHC16*. The four bp deletion (blue in a red box) in gibbon leads to a nonsense mutation (red). Note that there is a G to A transition (DNA polymorphism) in a stop codon of gibbon (TAA) compared with human/chimpanzee and other primates (TAG). **(B)** PCR analysis of gibbon *SIRH11/ZCCHC16*. Upper panel shows the schematic representation of primer design. Lower panel shows agarose gel electrophoresis profile in each primer set. The arrows represent expected band size. M, 100 bp and 1 kb ladder; Gor, gorilla; Hla, white-handed gibbon; Ssy, siamang; Rhe, rhesus macaque; Mar, common marmoset; Sol, solvent only (no DNA). **(C)** Southern blot analysis of *Hla* and *Ssy*. Left and right panels show the result of hybridization using *SIRH11* and *TYR* probes, respectively. The arrows indicate expected band size. Gor, gorilla; Hla, white-handed gibbon; Ssy, siamang; Rhe, rhesus macaque; Mar, common marmoset. **(D)** Amino acid sequence alignment of Chiroptera SIRH11/ZCCHC16. The blue asterisks in a red box indicate a common nonsense mutation in megachiroptera. The asterisks, colons and periods below the amino acids indicate identical, strongly and weakly similar residues among six species, respectively. **(E)** DNA alignments around the common TAA nonsense mutation (red).

In the case of the white-cheeked gibbon (*Nomascus leocogenys*: *Nle*), there is a four-base pair deletion leading to a frameshift and the subsequent emergence of a nonsense codon just after it (Figure 2A). We confirmed all of these sequence changes in another white-cheecked gibbon at a Japanese zoo. In the dN/dS ratio in the pairwise comparison among the hominoids, *SIRH11/ZCCHC16* was found to be highly constrained among humans, chimpanzees and gorillas (0.20–0.42), while the values of the gibbon (*Nle*) compared to humans and chimpanzees are higher (0.61–0.74), and those compared to the gorilla and orangutan are close to 1 (0.84–0.90). Although in this kind of approximate method, it is not possible to compare pairwise dN/dS values in a rigorous way, these results suggest that the truncated *Nle* ORF has been subjected to a lesser degree of purifying selection compared to other hominoid members having the full-length SIRH11/ZCCHC16 (Table 1A). It is also probable that the *Nle SIRH11/ZCCHC16* has lost some function by losing its CCHC RNA-binding domain (Rajavashisth et al., 1989; Curtis et al., 1997; Chen et al., 2003; Schlatter and Fussenegger, 2003; Narayanan et al., 2006; Matsui et al., 2007).

**Table 1:** **Pairwise dN/dS analyses on several lineages with truncated SIRH11/ZCCHC16**.

**dN/dS**	**1**	**2**	**3**	**4**	**5**	**6**	**7**	**8**	**9**	**10**	**11**	**12**	**13**	**14**	**15**
**A**															
1. Human															
2. Chimpanzee	0.309														
3. pygmy_chimpanzee	0.196	0.303													
4. Western_lowland_gorilla	0.202	0.424	0.3												
5. Sumatran_orangutan	0.453	0.713	0.512	0.705											
6. Northern_white-cheeked_gibbon_a	0.703	0.741	0.613	0.902	0.836										
**B**															
1. Black_flying_fox															
2. Large_flying_fox	1.545														
3. Big_brown_bat	0.715	0.686													
4. Little_brown_bat	0.66	0.625	0.615												
5. Brandts_bat	0.682	0.646	0.529	0.625											
6. Myotis_davidii	0.718	0.679	0.609	0.597	0.53										
**C**															
1. Azaras_owl_monkey															
2. Mas_night_monkey	0.090														
3. Common_marmoset	0.749	0.657													
4. Tufted_capuchin	1.914	1.395	0.988												
5. Bolivian_squirrel_monkey	0.985	1.058	0.795	1.035											
**D**															
1. Damara_mole-rat															
2. Naked_mole-rat	0.813														
3. Domestic_guinea_pig	0.687	0.505													
4. Long-tailed_chinchilla	0.861	0.711	0.599												
5. Degu	0.634	0.575	0.408	0.639											
6. Lesser_Egyptian_jerboa	0.435	0.378	0.353	0.407	0.514										
7. Ords_kangaroo_rat	0.557	0.594	0.43	0.556	0.527	0.454									
8. Prairie_vole	0.485	0.492	0.549	0.63	0.53	0.439	0.582								
9. Chinese_hamster	0.514	0.556	0.509	0.54	0.602	0.385	0.462	0.546							
10. Golden_hamster	0.579	0.533	0.491	0.583	0.558	0.467	0.524	0.587	0.52						
11. Prairie_deer_mouse	0.549	0.527	0.505	0.616	0.525	0.454	0.55	0.708	0.556	0.635					
12. House_mouse	0.516	0.437	0.432	0.484	0.462	0.359	0.48	0.435	0.375	0.332	0.443				
13. Norway_rat	0.453	0.442	0.362	0.414	0.451	0.372	0.557	0.472	0.488	0.34	0.519	0.442			
14. Upper_Galilee_mountains_blind_mole_rat	0.421	0.43	0.393	0.531	0.585	0.437	0.449	0.643	0.603	0.658	0.642	0.496	0.613		
15. Thirteen-lined_ground_squirrel	0.525	0.517	0.605	0.535	0.442	0.316	0.437	0.424	0.383	0.473	0.428	0.369	0.339	0.361	
**E**															
1. Chiru															
2. Bison_bison_bison	0.881														
3. Cattle	1.254	0.19													
4. Wild_yak	1.313	0.323	nd												
5. Water_buffalo	1.639	0.601	0.9	0.976											
6. Goat	1.13	0.655	0.864	0.913	1.077										
7. Sheep	0.627	0.613	0.751	0.787	0.652	0.259									
8. Mouflon	0.676	0.644	0.789	0.825	0.688	0.331	nd								
**F**															
1. Balaenoptera_acutorostrata_scammoni															
2. killer_whale	1.415														
3. bottlenosed_dolphin	0.949	0													
4. Yangtze_River_dolphin	0.844	0.4	0.331												
5. Sperm_whale	0.574	0.722	0.575	0.466											

*(A) Pairwise dN/dS analysis on Homonidae, (B) Pairwise dN/dS analysis on Chiropreta, (C) Pairwise dN/dS analysis on Platyrrhini, (D) Pairwise dN/dS analysis on Rodentia, Hystricognathi species are shown yellow. (E) Pairwise dN/dS analysis on Ruminantia, (F) Pairwise dN/dS analysis on Cetacea. The dN/dS values more than 0.80 or less than 0.21 are shown in red or blue, respectively. When the dS value is 0, it is impossible to calculate this value, therefore, indicated as nd*.

As the gibbons are a close relative of the hominids, including humans, we further analyzed two other species in the Hylobatidae family, the white-handed gibbon, *Hylobates lar: Hla*, and the siamang, *Symphalangus syndactylus*: *Ssy*, and found that normal *SIRH11/ZCCHC16* was absent from these two species. We set up PCR conditions using primers designed from the gibbon (*Nle*) DNA sequence. These primers worked well even in gorilla, macaque and marmoset samples but none of the expected bands were obtained from *Hla* or *Ssy* (Figure 2B, top and middle columns). The results were almost the same with primers designed from the conserved sequences between gibbon and human (F3, F4, R3, R4; Figure 2B, top and bottom columns), and we confirmed that the quality of their genomic DNA was good enough for PCR analysis by amplifying tyrosinase (*TYR*) and lactase (*LCT*) genes as controls (Figure S2). Finally, we performed Southern blot analysis using a gorilla PCR fragment (F1-R1: 544 bp) as a probe and confirmed that there was no corresponding band to *SIRH11/CCHC16* that appeared in the white-handed gibbon and siamang (Figure 2C), suggesting that a large deletion or profound structural change had occurred in these two gibbon species. All these results demonstrate that gibbons in at least three out of four genera do not possess the normal full-length SIRH11/ZCCHC16 ORF as a result of deletion/structural changes or the lack of a CCHC RNA-binding domain, supporting the notion that the gibbon *SIRH11/ZCCHC16* gene is not functional and instead has become a pseudogene.

In the case of megabats, the nonsense mutation of *SIRH11/ZCCHC16* is conserved between these two species, while it is intact in the four closest microbat species (Microchiroptera) (Figures 2D,E), indicating that this mutation occurred relatively recently. Except for the loss of the C-terminal CCHC domain, the megabat SIRH11/ZCCHC16 ORF looks well conserved without further nonsense or frameshift mutations. In this case, it is difficult to estimate whether the truncated ORFs are functional or not. Although the dN/dS value between two megabats exhibits 1.5, it is difficult to distinguish whether they have been subjected to positive or neutral evolution (Table 1B). However, it may simply reflect the fact that these two species are too close to examine in terms of the evolutionary relationship in this way, because both the dN and dS values are approximately 10 times lower than in the four microbat species. Among the Chiroptera, the values between the megabats to the microbats are slightly higher (0.63–0.72) than those of the microbats (0.53–0.63; Table 1B). Although, it is possible that they might possess certain functions, the function of the truncated megabat SIRH11/ZCCHC16 have already been changed or lost, or is on the way to either of these fates, while the microbat SIRH11/ZCCHC16 has undergone purifying selection.

### Mutations resulting in the loss of the N-terminal half of the ORF in the boreotherians

**Figure 3:**
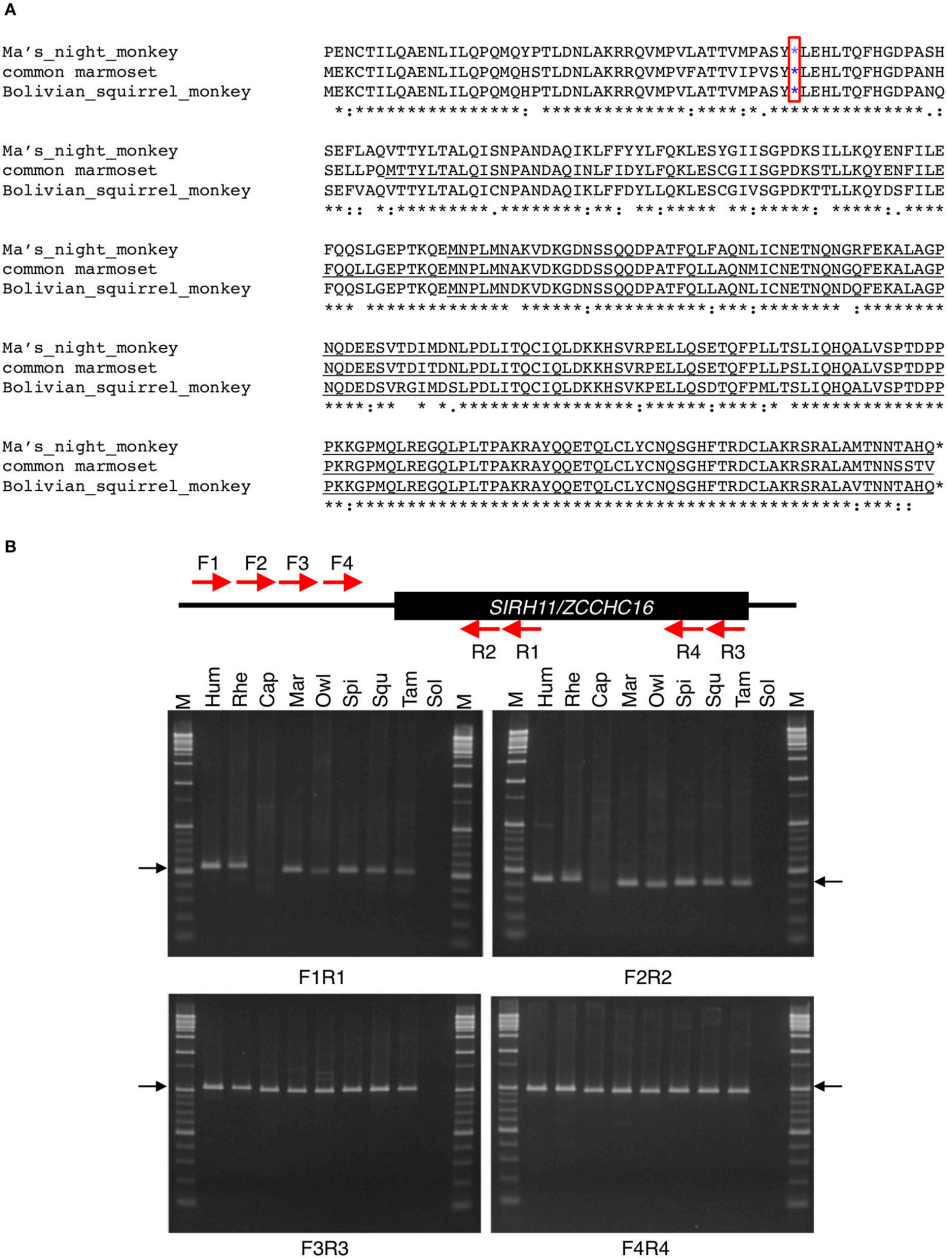

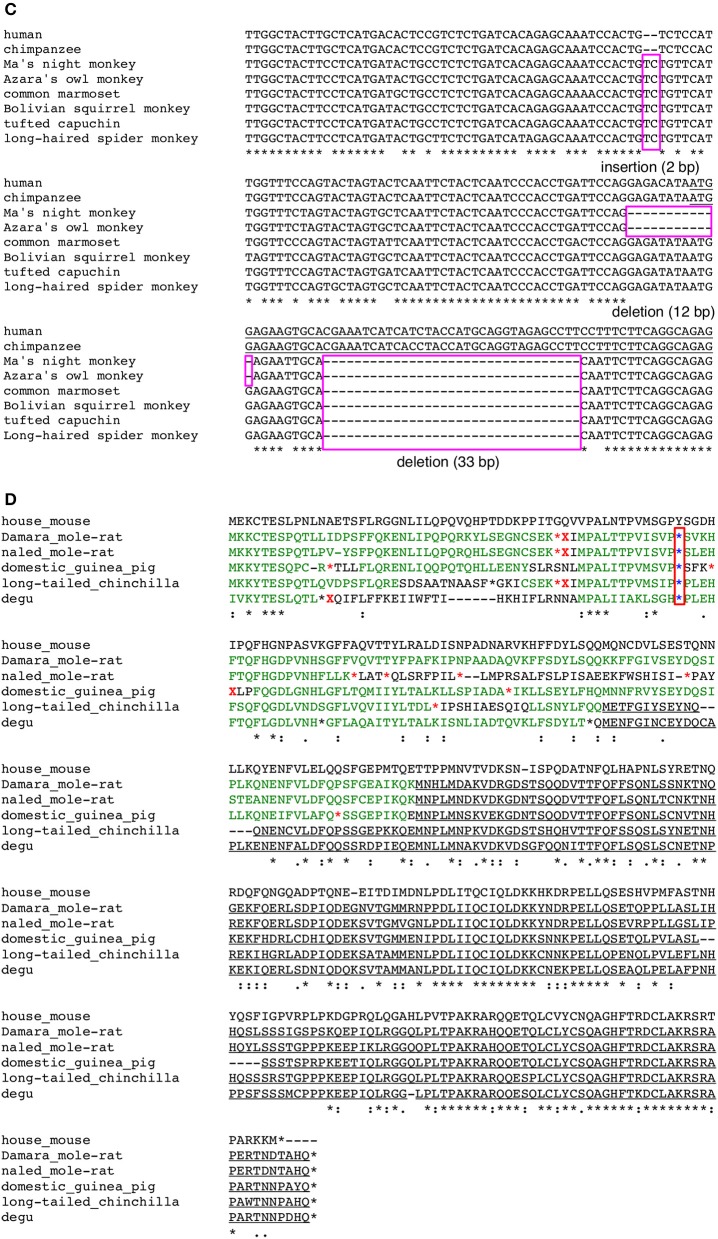

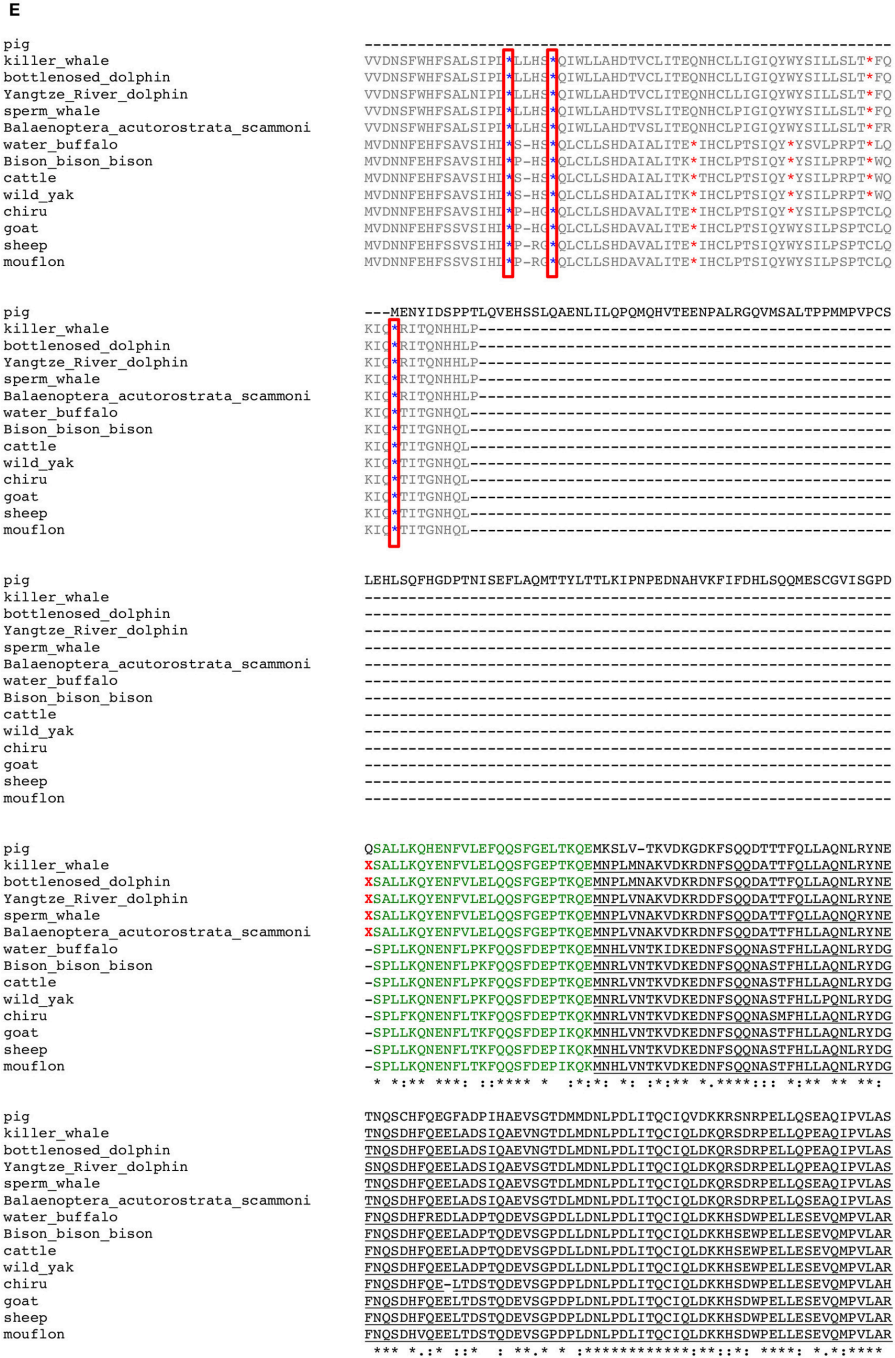

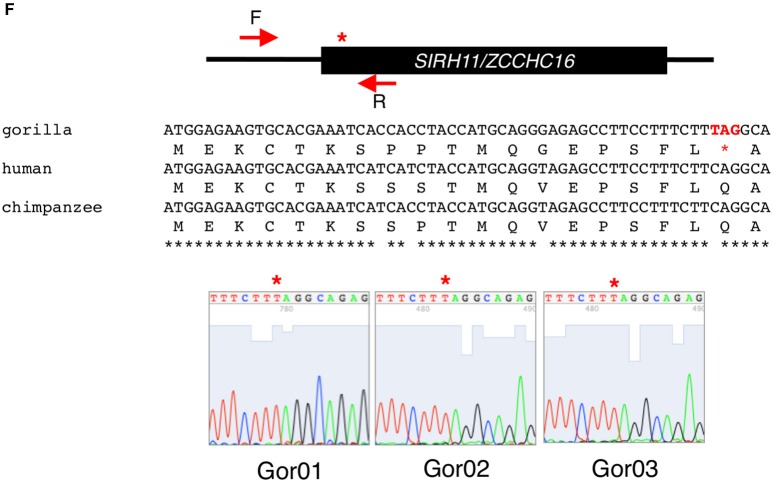
**(A)** Amino acid sequence alignment of Platyrrhihi *SIRH11/ZCCHC16*. The blue asterisks indicate common nonsense mutations in Platyrrhihi. The underlined sequences indicate the putative short ORFs starting from a next Met codon. The asterisks, colons and periods below the amino acids indicate identical, strongly and weakly similar residues among three species, respectively. **(B)** PCR analysis of Platyrrhihi *SIRH11/ZCCHC16*. Upper panel shows the schematic representation of the primer design. Lower panel shows agarose gel electrophoresis profile in each primer set. The arrows represent expected band size. M, 100 bp and 1 kb ladder; Hum, human; Rhe, rhesus macaque; Cap, tufted capuchin; Mar, common marmoset; Owl, the Azara's owl monkey; Spi, long-haired spider monkey; Squ, common squirrel monkey; Tam, cotton-top tamarin; Sol, solvent only (no DNA). **(C)** DNA sequence analysis of Platyrrhihi *SIRH11/ZCCHC16*. DNA sequences of Azara's owl monkey, common marmoset, tufted capuchin, and long-haired spider monkey determined by our own experiments are also shown. Magenta boxes show lineage specific insertion or deletion. The underlined letters indicate the *SIRH11/ZCCHC16* ORF. **(D)** Amino acid sequence alignment of Hystricognathi SIRH11/ZCCHC16. The similar sequences among five Hystricognathi species are expressed in green. The red asterisks and Xs indicate the sites of ORF termination and frameshift, respectively. The blue asterisks in a red box indicate a common nonsense mutation in Hystricognathi. The underlined sequences indicate the putative short ORFs starting from a next Met codon. The asterisks, colons and periods below the amino acids indicate identical, strongly and weakly similar residues among six species, respectively. The house mouse sequence is used as a reference. **(E)** Amino acid sequence alignment of Cetartiodactyla SIRH11/ZCCHC16. The blue asterisks in red boxes indicate common nonsense mutations in Cetartiodactyla. The red asterisks and Xs indicate the sites of ORF termination and frameshift, respectively. The underlined sequences indicate the putative short ORFs starting from a next Met codon. The similar sequences among 14 Cetartiodactyla species are shown in green. The asterisks, colons and periods below the amino acids indicate identical, strongly and weakly similar residues among 14 species, respectively. The pig sequence is used as a reference. **(F)** Sequence analysis of gorilla *SIRH11/ZCCHC16*. Upper panel shows the schematic representation of the primer design and nonsense mutation site (red asterisk) in gorilla *SIRH11/ZCCHC16*. Middle panel shows the sequence comparison between gorilla, human, and chimpanzee. Lower panel represents the sequence results of three individuals.

In three lineages in boreotheria, a deletion of the N-terminal half of SIRH11/ZCCHC16 ORF was observed [i.e., the New World monkeys (Primates: three species/three examined (3/3)), the New World and African hystricognaths (Rodentia: 3/3 and 2/2, respectively) as well as species belonging to Cetacea and Ruminantia (Cetartiodactyla: 5/5 and 7/7, respectively)] (Figure 3). In all of these species, short putative ORFs, mainly comprising 167 aa, are conserved although the causative mutations are independent of each other, reflecting their own lineage-specific events. In these cases, the pairwise dN/dS analyses suggest that there has been selective constraint in some species and perhaps a more relaxed or neutral selection in others.

Among three New World monkeys, Ma's night monkey, the Bolivian squirrel monkey and common marmoset, a common deletion of 11 aa was observed near the N-terminus, with a common nonsense mutation just after it (Figures 3A,C). The putative short ORF that starts from the next Met codon comprises 167 aa in the first two and 241 aa in the latter because of a single additional Met codon that arose in a species-specific manner (Figure S1). We further analyzed five more species, the long-haired spider monkey (*Ateles belzebuth: Abe*), common squirrel monkey (*Saimiri sciureus: Ssc*), tufted capuchin (*Cebus apella*: Cap), the Azara's owl monkey (*Aotus azarae: Aaz*) and the cotton-top tamarin (*Saguinus oedipus: Soe*) and confirmed the common deletion of 11 aa and the subsequent nonsense mutation are a lineage-specific feature (Figures 3B,C). Thus, it seems probable that these mutations emerged in the common Platyrrihini ancestor from which all the New World monkeys in South America diverged (Poux et al., 2006).

A similar situation was assumed in the case of the two closest rodent groups, the South American and African hystricognaths (Caviomorpha and Bathyergidae; Poux et al., 2006). The lost N-terminal parts contain several nonsense mutations and frameshifts, and only one nonsense mutation is conserved in all of the species (Figure 3D), indicating that this mutation emerged in a common ancestor in Africa (Poux et al., 2006). Compared with the heavily mutated N-terminal region, the C-terminal region, comprising 161–203 aa, is highly conserved.

Compared with the above two cases, DNA sequences corresponding to the N-terminal half are completely missing in the species of Cetacea and Ruminantia, indicating a large deletion occurred in the common ancestor of these two suborders, although the former has an additional frameshift event that took place in a Cetacea-specific manner (Figure 3E, Figure S3). The remaining ORFs, each comprising 167 aa, are also highly conserved.

The pairwise dN/dS analysis within each lineage suggests that some portions of the tree display gene-wide dN/dS values that are consistent with purifying selection. For example, among five New World monkey species the truncated SIRH11/ZCCHC16 ORFs are highly constrained (dN/dS = 0.09) between the two night monkeys (the Azaras owl monkey and Ma's night monkey; Table 1C). However, those of the tufted capuchin are variable: greater than 1 (1.9 and 1.4) to the night monkeys, close to or less than 1 (0.99 and 0.80) to the common marmoset and Bolivian squirrel monkey. Those of the Bolivian squirrel monkey are consistently close to 1 (0.80–1.1), suggesting neutral evolution. Thus, it is possible that the functions of the truncated ORFs in Platyrrhini were diversified to a great extent, presumably because of species-specific adaptation after the structural change of N-terminus deletion in the common Platyrrhini ancestor.

In the case of rodents, the truncated SIRH11/ZCCHC16 ORFs in Hystricognathi are highly constrained among Rodentia except for the Damara mole rat and naked mole rat, and the Damara mole rat and long-tailed chinchilla (0.81 and 0.86, respectively; Table 1D). The cases of the Ruminantia (Table 1E) and Cetacea (Table 1F) are similar to the Platyrrhini in that the dN/dS values exhibit a large variety, some are highly constrained (between bison and cattle, as well as sheep and goats) while the others seem to be subjected to neutral evolution. All these results suggest that the function of the truncated SIRH11/ZCCHC16 ORFs diverged in a species-specific manner, possibly reflecting the functional constraints imposed by the environment after the lineage-specific structural changes.

Some species have ORFs with a small deletion of the N-terminal region, such as the western lowland gorilla, with a 28 aa deletion (Primates), and the upper Galilee mountains blind mole rat, with a 22 aa deletion (Rodentia). In the former, using three different individuals we confirmed the nonsense mutation close to the translational start site caused by a frameshift (Figure 3F).

### Loss of *SIRH11/ZCCHC16* in xenathrans

We previously reported that *SIRH11/ZCCHC16* in two armadillo species (*Dasypus novemcinctus* and *Tolypeutes matacus*: Cingulata) and two sloth species (*Choloepus hoffmanni* and *Choloepus didactylus*: Pilosa) were pseudogenized due to severe mutations, including multiple nonsense mutations and frameshifts (Irie et al., 2015). There are no common mutations between two armadillo species belonging to two different genera, although the genomic DNA information corresponding to the C-terminal part was lacking in GenBank for one armadillo species (*Dasypus novemcinctus*). Two sloth species belonging to the same genera exhibit a quite similar pattern of nonsense and frameshift mutations.

**Figure 4:**
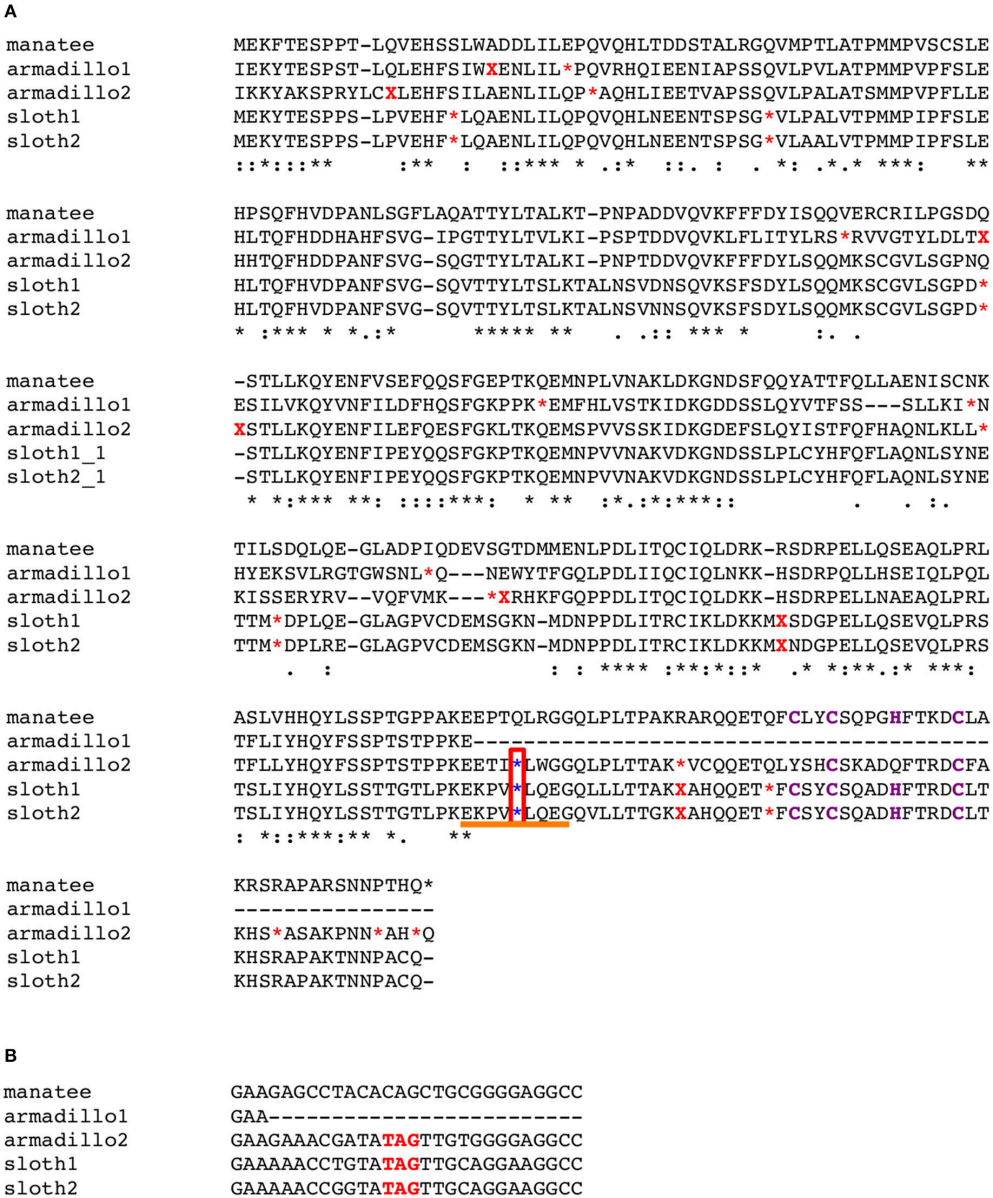

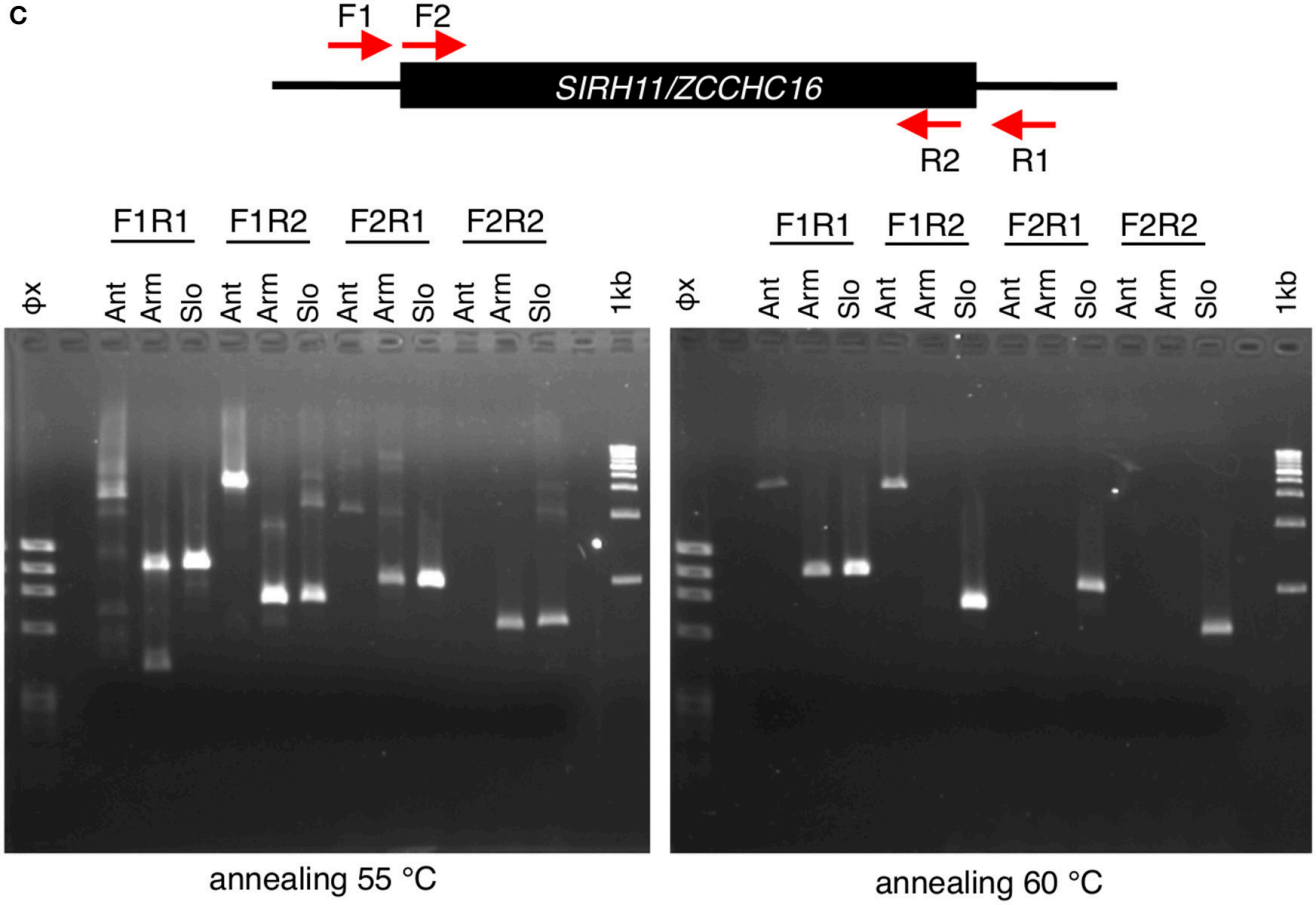
**(A)** Common nonsense mutation among xenarthra species. Multiple sequence alignment was constructed using five amino acid sequences; armadillo1, *Dasypus novemcinctus*; armadillo2, *Tolypeutes matacus*; sloth1, *Choloepus hoffmanni*; sloth2, *Choloepus didactylus* and manatee, Florida manatee as a reference. The red asterisks and Xs show the sites of ORF termination and frameshift, respectively. The blue asterisks in a red box indicate a common mutation among three species. Purple characters indicate CCHC amino acids in the RNA binding domain. **(B)** DNA alignments around the common TAG nonsense mutation (red) indicated by an orange line in **(A)**. **(C)** PCR analysis of xenarthra *SIRH11/ZCCHC16* Upper panel: schematic representation of primer design to amplify xenarthra *SIRH11/ZCCHC16*. Lower panel: agarose gel electrophoresis profile in each primer set. Ant, giant anteater; Arm, southern three-banded armadillo; Slo, Linnaeus's two-toed sloth; Φx, Φx174 *Hae*III marker; 1 kb, 1 kb ladder marker.

Then, we searched for a common nonsense mutation in another armadillo species (*Tolypeutes matacus*) along with the two sloth species again and found a promising candidate nonsense mutation in the C-terminus, 22 aa upstream of the CCHC RNA-binding domain (Figure 4A, blue asterisks in a red box, and Figure 4B). This pattern of a shared nonsense mutation is consistent with the possibility that this mutation inactivated the gene, because the CCHC domain would be critical for normal SIRH11/ZCCHC16 function, as in the case of gibbons mentioned above. In this work, we have surveyed the mutations only in the ORF region, but it should be noted that pseudogenization may have occurred through promoter or other regulatory mutations and also from a missense mutation or insertions/deletions that inactivates the gene.

The anteater is the only remaining animal group in xenarthra, belonging to Pilosa, the same order as the sloth (Delsuc and Douzery, 2009). An attempt was made to analyze *SIRH11/ZCCHC16* in the giant anteater (*Myrmecophaga tridactyla*) by genomic PCR, but the PCR did not work, even using primer sets designed with completely conserved DNA regions between armadillos (Cingulata) and sloths (Pilosa) (Figure 4C). Although, a single band larger than an expected size was seen in the two conditions (Figure 4C, F1R1, and F1R2), its DNA sequence did not have any relationship to *SIRH11/ZCCHC16* (data not shown). The quality of its genomic DNA was good enough for PCR, as shown by amplifying the tyrosinase gene (*TYR*) using primers designed from the armadillo DNA sequence (Figure S4). However, due to the limited amount and relatively low quality we were unable to perform Southern blot analysis to confirm the absence of *SIRH11/ZCCHC16* orthologs in its genome. Therefore, the final conclusion awaits the determination of the anteater genome sequence in the future, but all of the results obtained thus far suggest that the three extant groups in xenarthra lack any functional *SIRH11/ZCCHC16* and that the pseudogenizing mutation(s) occurred in a common xenarthran ancestor.

## Discussion

It is of interest to determine the roles genes acquired from LTR retrotransposons play in organisms in the current form of the developmental system as well as in the course of biological evolution. Among *SIRH* genes, *PEG10/SIRH1, PEG11/RTL1/SIRH2*, and *SIRH7/LDOC1* are highly conserved across eutherian species, presumably because they play essential roles in the viviparous reproduction system via placental formation, maintenance, differentiation and maturation, respectively (Ono et al., 2006; Sekita et al., 2008; Naruse et al., 2014). In this study, we found that *SIRH11/ZCCHC16* displays lineage-specific structural variations in eutherians, such as the lack of the CCHC RNA-binding domain or the N-terminal half, as well as species-specific variations in the resulting truncated ORFs. Thus, it is possible that certain *SIRH* genes, such as those concerning cognitive brain function, act as critical determinant factors in the diversification of the eutherians depending on a variety of environmental factors, such as ecological niches and the dynamics of life style as well as the evolutionary history of the species, including geological events.

We showed that all the South American primates and rodents have the truncated SIRH11/ZCCHC16 ORFs in addition to xenarthran *pseudoSIRH11/ZCCHC16*. Although this might not be of significance, it is of interest to consider the possibility that species with normal *SIRH11/ZCCHC16* function suffered a competitive disadvantage in the South American environment in the past. South America has a unique evolutionary history in which geographical factors have played a critical role (Houle, 1999; Poux et al., 2006; Delsuc and Douzery, 2009; Murphy and Eduardo, 2009; Nishihara et al., 2009). Diversification of the three major eutherian groups, boreotheria, afrotheria and xenarthra, is supposed to largely be dependent on the division of the supercontinent Pangea, which is thought to have occurred approximately 120 Mya (Nishihara et al., 2009). Xenarthrans evolved and diverged on the isolated South American continent, where carnivorous marsupials and birds had long predominated (Patterson and Pascual, 1972; Murphy and Eduardo, 2009). After the Isthmus of Panama emerged ~3 Mya, the carnivorous marsupials were replaced by an invading carnivorous laurasiatherian species from North America (Patterson and Pascual, 1972). In the competition between the carnivorous marsupials and xenarthrans after the extinction of the dinosaurs ~65 Mya, as well as the marsupials and the carnivorous laurasiatherians ~3 Mya, the presence/absence of *SIRH11/ZCCHC16* among these groups might be a critical factor in the evolutionary outcome. For example, the extinct marsupials have no *SIRH11/ZCCHC16*, the xenarthrans have *pseudoSIRH11/ZCCHC16* and only the laurasiatherians have a normal *SIRH11/ZCCHC16* in the South American evolutionary history.

Another issue of interest is the *SIRH11/ZCCHC16* mutations in the primates. Phylogenetic relationships, divergence times, and patterns of biogeographic descent among primate species are complex and still controversial. According to a recent molecular phylogenetic analysis using Species Supermatrix, the currently living primates last shared a common ancestor 71–63 Mya and Asia was the ancestral home of the primates. This is also true for the hominoids, suggesting that the ancestor of African apes and humans entered Africa, while the hylobatids remained in Asia (Springer et al., 2012). Among the hominoids, gibbons have lost the normal *SIRH11/ZCCHC16*: white-cheeked gibbon (*Nle*) has a truncated SIRH11/ZCCHC16 ORF lacking the CCHC RNA-binding domain, while white-handed gibbon (*Hla*) and siamang (*Ssy*) do not have the normal *SIRH11/ZCCHC16* gene in their genomes. It is apparent that *SIRH11/ZCCHC16* function is not conserved in the latter two species, but is this the case for the truncated SIRH11/ZCCHC16 in the former? It is typically difficult to determine whether truncated ORFs have original, similar or different functions through comparison of the amino acid sequence homology. The dN/dS analysis sometimes helps provide a useful prediction of whether they still possess some function (dN/dS < 1) or have lost their function and already become neutralized (dN/dS = 1), as previously shown in the xenarthran lineage (Irie et al., 2015). The higher dN/dS values of the gibbon (*Nle*) suggested that the truncated *Nle* ORF has been subjected to a lesser degree of purifying selection and that the *Nle SIRH11/ZCCHC16* has lost some function by losing its CCHC RNA-binding domain. In retroviruses, the CCHC domain forms a part of the nucleocapsid protein that functions in virus genome packaging and the early infection process (Narayanan et al., 2006). Proteins containing the CCHC zinc-finger domain are commonly known to interact with single-stranded DNAs (ssDNAs) and RNAs (Matsui et al., 2007) and play important roles in *Drosophila* as well as mammalian development via transcriptional and translational regulations (Rajavashisth et al., 1989; Curtis et al., 1997; Chen et al., 2003; Schlatter and Fussenegger, 2003). Therefore, it is probable that the CCHC zinc-finger domain is essential for the normal SIRH11/ZCCHC16 function that confers selective advantage. In future, it will be of interest to consider the possibility that *SIRH11/ZCCHC16* contributed to brain evolution in hominoidea and also the alternative, that the loss of *SIRH11/ZCCHC16* did confer some selective advantage in the gibbons.

Lineage-specific loss of the N-terminus of SIRH11/ZCCHC16 ORFs in all the species of New World monkeys and Hystricognathi is consistent with their evolutionary history. Our data indicates a common ancestor of the Platyrrhini in South America already had the mutation(s) leading to the N-terminal deletion. It is proposed that the common ancestor emigrated from Africa and somehow immigrated into South America ~34 Mya (Houle, 1999; Poux et al., 2006), possibly by an incidental current drift from Africa to South America that existed at that time (Houle, 1999). In the case of the two closest South American and African hystricognaths, the Caviomorpha and Bathyergidaein, the results also show that the nonsense mutation leading to the N-terminal deletion first emerged in the common ancestor in Africa. It is proposed that a common Caviomorpha ancestor, from which all the rodent species in South America diverged, emigrated from Africa by an unknown event, just as New World monkeys did (Houle, 1999). The recent discovery of (Late) Eocene primates in Santa Rosa, Peru, extends the fossil record of primates in South America back approximately 10 million years, leading to consideration of possible similarities of an intercontinental dispersal mechanism for the two mammalian groups that occurred around 36 Mya (Bond et al., 2015). However, the Eocene primates bear little resemblance to any extinct or living South American primates, but do bear striking resemblance to Eocene African anthropoids while the Santa Rosa rodents exhibit the derived status relative to the contemporaneous African rodents. Then, these authors suggested two possibilities that rodents and primates might not have had simultaneous crossing episodes or that the two groups had differing rates of diversification after their arrival in South America (Bond et al., 2015). Our results appear to support the latter idea, because the patterns of Hystricongathi and Platyrrhini *SIRH11/ZCCHC16* diversification are very different, i.e., conservative vs. highly diversified, although this might not be directly related to the morphology of the molars.

In the Chiroptera, the dN/dS analysis did not provide good evidence to indicate that *SIRH11/ZCCHC16* is subject to different types of evolutionary selection between the megabats and microbats. This may be because the numbers of species are limited, resulting in the fact that a more detailed analysis is necessary to construct a precise evolutionary view among such closely related species. It is known that between these two suborders of Chiroptera, sophisticated laryngeal echolocation system is absent in Megachiroptera (Teeling, 2005, 2009). Therefore, it will be of interest to elucidate how structural changes in SIRH11/ZCCHC16 relate to certain neurological changes affecting differences in this behavior between these two suborders of Chiroptera. It should be noted that recent molecular data indicate that Microchiroptera is not a monophyletic group, thus, suggesting that sophisticated laryngeal echolocation in the bats either originated in the ancestor of all bats and was subsequently lost in lineages leading to the megabats or originated more than once in the microbat lineages (Teeling et al., 2000). We found that the dN/dS values exhibit a large variety in several eutherian lineages that display the N-terminus deletion. This finding suggests that the function of the truncated SIRH11/ZCCHC16 ORFs diverged in a species-specific manner, implying that the protein contributed to diversification of eutherians by increasing evolutionary fitness although *SIRH11/ZCCHC16* itself is not an essential gene in eutherian development and growth. However, it will be necessary to carry out maximum likelihood estimates of the dN/dS values using PAML branch models or other techniques to obtain supportive evidence for this idea.

Knockout mice demonstrated that *Sirh11/Zcchc16* is involved in cognitive function, including attention, impulsivity and working memory. In mice, *Sirh11/Zcchc16* is expressed in the adult kidney, testis and ovary in addition to the brain, but male and female KO mice exhibited normal fertility and kidney function. However, it is possible that it also plays some role in the kidney, testis, ovary and embryonic liver where *Sirh11/Zcchc16* expression was confirmed. Human *SIRH11/ZCCHC16* is expressed in similar tissues and organs, such as the adult brain, liver, kidney and testis, as shown by RT-PCR, although the levels are very low, as in the case of mice (Figure S5). Therefore, it is important to identify the roles of *SIRH11/ZCCHC16* in some other organs rather than brain in different lineages and species. It is of particular interest also to determine its function in humans because of X-linked intellectual disability and attention-deficit/hyperactivity linked phenotypes of the *Sirh11/Zcchc16* knockout mice.

## Materials and methods

### Ethics

All experiments using primate samples were performed in Kyoto University Primate Research Institute (KUPRI), in accordance with the Guidelines for Care and Use of Nonhuman Primates (Version 3; June 2010) published by KUPRI. For usage of these samples and publication of the results, we obtained permissions from the respective zoos that provided the samples.

### Primate samples

All hominoid DNA samples were extracted from liver pieces collected from animals that died of natural causes at zoos except *Nomascus leocogenys* (*Nle*: white-cheeked gibbon). *Nomascus* genomic DNA was isolated from its feces provided by Hirakawa Zoological Park. All New World monkey DNA samples were extracted from cultured epithelial cells originating from animals bred at KUPRI. The cultured cells were derived from a tiny piece of the ear skin of a live animal anesthetized for other purposes, such as a medical treatment or health checkup.

### PCR analysis

For gibbon *SIRH11* analysis, we prepared genomic DNA of *Hylobates lar* (*Hla*: white-handed gibbon) and *Symphalangus syndactylus* (*Ssy*: siamang). The PCR reaction was performed using PrimeSTAR GXL DNA Polymerase (TaKaRa, Japan) with the following conditions: 94°C, 2 min; 4 cycles of 98°C, 10 s, 50°C, 15 s, 68°C, 1 min; 32 cycles of 98°C, 10 s, 56°C, 15 s, 68°C, 15 s; final extension 68°C, 1 min. The following PCR primers were used: gibbon_SIRH11_F1: 5′- GGCATCTCTCCAATTCAGCTGTTAGCAACT-3′, gibbon_SIRH11_R1: 5′- GGCAAGGCAATCTCTTGTGAAGTGACC ACA-3′, gibbon_SIRH11_F2: 5′- AGTGTCTTCTTCACAGCTAACAGCTTTGGC-3′, gibbon_SIRH11_R2: 5′- CTGCAGTAGAGGCACAAATGAGTTTCT AGC-3′, gibbon_SIRH11_F3: 5′- ACATATCTGGGCCTGACAAGAG-3′, gibbon_SIRH11_R3: 5′- GGCTTGGTGTTGGATCAAGG-3′, gibbon_SIRH11_F4: 5′- AGCAGTCATTTGGTAAACCCAC-3′, gibbon_SIRH11_R4: 5′- CAAGGAAGCCAACAATGGGAG-3′.

For Platyrrhini *SIRH11* analysis, we prepared genomic DNA of five species: Tufted capuchin (Cap), common marmoset (Mar), the Azara's owl monkey (Owl), long-haired spider monkey (Spi), common squirrel monkey (Squ) and cotton-top tamarin (Tam). Human (Hum), rhesus macaque (Rhe) DNAs were used as controls. The PCR reaction was performed using PrimeSTAR GXL DNA Polymerase (TaKaRa, Japan) with following conditions: for F1R1 and F2R2 primer sets, 94°C, 2 min; 4 cycles of 98°C, 10 s, 50°C, 15 s, 68°C, 2 min; 30 cycles of 98°C, 10 s, 64°C, 15 s, 68°C, 40 s; final extension 68°C, 1 min; for F3R3 and F4R4 primer sets: 94°C, 2 min; 4 cycles of 98°C, 10 s, 50°C, 15 s, 68°C, 1 min; 30 cycles of 98°C, 10 s, 56°C, 15 s, 68°C, 15 s; final extension 68°C, 1 min. The following PCR primers were used: Platyrrhini_SIRH11_F1: 5′-GGCATCTCTCCAATTCAGCTGTTAGCAACT-3′, Platyrrhini_SIRH11_F2: 5′-AGTGTCTTCTTCACAGCTAACAGCTTTGGC-3′, Platyrrhini_SIRH11_F3: 5′-GAGGGAGGAGAGAAAGGTACTG-3′, Platyrrhini_SIRH11_F4: 5′-TGCAGAACATTGGCCTTTTCC-3′, Platyrrhini_SIRH11_R1: 5′-GGCAAGGCAATCTCTTGTGAAGTGACCACA-3′, Platyrrhini_SIRH11_R2: 5′-CTGCAGTAGAGGCACAAATGAGTTTCTAGC-3′, Platyrrhini_SIRH11_R3: 5′-TCTGAGCAATTGGCAGGGTC-3′, Platyrrhini_SIRH11_R4: 5′-GGTCACCATGAAACTGGGTG-3′. The PCR products were directly-sequenced.

For gorilla *SIRH11* analysis, genomic DNA was isolated from frozen liver. The PCR reaction was performed using PrimeSTAR GXL DNA Polymerase (TaKaRa, Japan) with the following conditions: 94°C, 2 min; 36 cycles of 98°C, 10 s; 55°C, 15 s; 68°C, 20 s; final extention 68°C, 60 s. The following PCR primers were used: Gorilla_SIRH11_F1: 5′-GAGGGAGGAGAGAAAGGTACTG-3′ and Gorilla_SIRH11_R1: 5′-TCTGAGCAATTGGCAGGGTC-3′.

For xenarthra *SIRH11* analysis, we prepared genomic DNA from three xenarthran species: *Tolypeutes matacus* (southern three-banded armadillo), *Choloepus didactylus* (Linnaeus's two-toed sloth), and *Myrmecophaga tridactyla* (giant anteater). Genomic DNA was isolated from frozen tissues using the DNeasy Blood & Tissues Kit (QIAGEN, Germany). The PCR reaction was performed using Ex*Taq*HS (TaKaRa, Japan) with the following conditions: 96°C, 3 min; 30 cycles of 98°C, 10 s; 55 or 60°C, 30 s; 72°C, 60 s; final extension 72°C, 3 min. The following PCR primers were used: Xenarthra_SIRH11_F1: 5′-CTTACTGCCTGCCCATTGGT-3′, Xenarthra_SIRH11_R1: 5′-GGATTTTAAAAGTTGGTGCAGG-3′, Xenarthra_SIRH11_F2: 5′-GGCAGAGAATCTGATTCTA-3′, Xenarthra_SIRH11_R2: 5′-GTATTGGTGGTAGATCAGG-3′.

### DNA sequencing of *SIRH11/ZCCHC16* in primate species

The Gorilla_SIRH11_F1R1 PCR products described above were cloned into the pBluescript II SK (+) vector and sequenced using a forward primer: 5′-TGTAAAACGACGGCCAGT-3′ and a reverse primer: 5′-CAGGAAACAGCTATGACCATG-3′. Platyrrhini_SIRH11_F1R1 PCR products described above were directly-sequenced using Platyrrhini_SIRH11_F1 and R1 primers. DNA Data Bank of Japan (DDBJ) accession numbers: LC150703 for western lowland gorilla *SIRH11/ZCCHC16*, LC150704 for Tufted capuchin, LC150705 for the Azara's owl monkey and LC150706 for long hair spider monkey *SIRH11/ZCCHC16*.

### Southern blot analysis

We prepared genomic DNA of five species: gorilla (Gor), *Hylobates lar* (Hla: white-handed gibbon), *Symphalangus syndactylus* (Ssy: siamang), rhesus macaque (Rhe), and marmoset (Mar). Twelve microgram of genomic DNA were digested by restriction enzymes, *Hind*III and *Xba*I. Southern blot analysis was performed using standard protocol (electrophoresis: submerged in 1x TAE buffer, at 1.2 V/cm, for 18 h at 4°C using 1.2% agarose gel; Treatment of DNA in gel: denaturation with 0.5 N NaOH/0.5 M NaCl for 30 min at 20–30°C, neutralization with 0.5 M Tris/0.5 M NaCl (pH7.0) for 15 min at 20–30°C; Capillary transfer to nylon membrane, Hybond-N+ (GE Healthcare): 5x SSC was supplied and absorbed by paper stack, for 4 h at 20–30°C; hybridization: 59°C for 12.5 h). The *TYR* and *SIRH11* probes were generated by genomic PCR using gorilla DNA as a template, respectively. The probe labeling, hybridization, washes, and detection were performed using AlkPhos System (GE Healthcare), per manufactures protocol.

### Computational analysis

Eighty-five eutherian mammal *SIRH11* genome sequences were downloaded from NCBI (http://www.ncbi.nlm.nih.gov/). Two *SIRH11* sequences we previously identified in xenarthra species, *Tolypeutes matacus* and *Choloepus didactylus*, were obtained from DDBJ accession LOC064756 and LOC064757, respectively. The *SIRH11* ORF in each species was identified by NCBI nucleotide blast search (http://blast.ncbi.nlm.nih.gov/Blast.cgi) using human *SIRH11* ORF sequence (GenBank Accession No. NC_000023: 112454729-112455661) as the query sequence. EMBOSS Transeq (http://www.ebi.ac.uk/Tools/st/emboss_transeq/) was used for translation nucleotide sequence to amino acids sequence. Multiple sequence alignment was constructed using Clustal Omega (http://www.ebi.ac.uk/Tools/msa/clustalo/) in the default mode.

### Estimation of the dN/dS ratio

An amino acid sequence phylogenic tree was constructed with MEGA6 (Tamura et al., 2013) using Maximum Likelihood method based on the JTT matrix based model. The codon alignment of cDNA was created with the PAL2NAL program (www.bork.embl.de/pal2nal/) (Suyama et al., 2006). The nonsynonymous/synonymous substitution rate ratio (ω = dN/dS) was estimated by using CodeML (runmode: −2) in PAML (Yang, 2007).

## Author contributions

Conceived and designed the experiments: TK, FI. Performed the experiments: MI, AK. Analyzed the data: MI, AK, TK, FI. Analyses of primate samples including DNA sequencing, genomic PCR and Southern blot analysis: AK. Wrote the paper: TK, FI.

## Funding

This work was supported by funding program for Next Generation World-Leading Researchers (NEXT Program) from the Japan Society for the Promotion of Science (JSPS) to TK, Grants-in-Aid for Scientific Research 15H04427 to AK, Grants-in-Aid for Scientific Research (S) from the Ministry of Education, Culture, Sports, Science, and Technology (MEXT) of Japan to FI, TK.

### Conflict of interest statement

The authors declare that the research was conducted in the absence of any commercial or financial relationships that could be construed as a potential conflict of interest.

## Supplementary material

Figure S1 **Alignment of amino acid sequences of SIRH11/ZCCHC16 from 83 eutherian mammals (related to Figure 1)**.

Figure S2 **PCR analysis of gibbon *TYR* and *LCT* (related to Figure 2B)**. Left panel shows the scheme of PCR primer design. Right panel shows agarose gel electrophoresis profile in each primer set. The arrows represent expected band size. To confirm the quality of genomic DNAs of *Hla* and *Ssy*, we amplified tyrosinase (*TYR*) and lactase (*LCT*) genes using the PCR primers designed on the basis of the human sequence. For *TYR* gene amplification, the PCR reaction was performed using PrimeSTAR GXL DNA Polymerase (TaKaRa, Japan) with the following conditions: 94°C, 2 min; 4 cycles of 98°C, 10 s, 50°, 15 s, 68°C, 2 min; 30 cycles of 98°C, 10 s, 64°C, 15 s, 68°C, 40 s; 68°C, 1 min. The following PCR primers were used: human_TYR_F: 5′-TAAGAGAAGCTCTATTCCTGACACTACCTC-3′ and human_TYR_R: 5′-AGCTGGTGCTTCATGGGCAAAATCAATGTC-3′. For *LCT* gene amplification, the PCR reaction was performed using PrimeSTAR GXL DNA Polymerase (TaKaRa, Japan) with the following conditions: 94°C, 2 min; 4 cycles of 98°C, 10 s, 54°C, 15 s, 55°C, 2 min; 30 cycles of 98°C, 10 s, 64°C, 15 s, 68°C, 50 s; 68°C, 1 min. The following PCR primers were used: human_LCT_F: 5′-AGTTCGAAAGAGATTTGTTCTACCACGGGA-3′ and human_LCT_R: 5′-AGCTCTGTTCATTGCCGTGGAAGGCCACGA-3′.

Figure S3 **DNA sequence alignment of Cetartiodactyla *SIRH11/ZCCHC16* (related to Figure 3E)**. Among 14 species, corresponding DNA sequences upstream and around the first Met of pig SIRH11/ZCCHC16 and those of the short ORFs of the other 13 Cetartiodactyla species are highly conserved, demonstrating that a large deletion occurred in this lineage.

Figure S4 **PCR analysis of xenarthran *TYR* (related to Figure 4B)**. Left panel shows the scheme of PCR primer design. The PCR primers, F1 and R1, were designed in consensus sequences between the armadillo (*Dasypus novemcinctus*) and sloth (*Choloepus hoffmanni*) *TYR*. Right panel shows agarose gel electrophoresis profile in each primer set. The arrows represent expected band size. To confirm the quality of anteater genomic DNA, we amplified the tyrosinase (*TYR*) gene in three xenarthra speicies, *Tolypeutes matacus* (southern three-banded armadillo), *Choloepus didactylus* (Linnaeus's two-toed sloth) and *Myrmecophaga tridactyla* (giant anteater). The PCR reaction was performed using Ex*Taq*HS (TaKaRa, Japan) with the following conditions: 96°C, 3 min; 30 cycles of 98°C, 10 s; 55°C, 30 s; 72°C, 60 s; final extension 72°C, 3 min. The following PCR primers were used: Xenarthra_TYR_F1: 5′-GTTAGTCATGTGCTTTTCAGAAG-3′ and Xenarthra_TYR_R1: 5′-CCAGGTGCTTCATGAGCAAAAT-3′.

Figure S5 **Expression of human *SIRH11/ZCCHC16* in adult tissues and organs**. Upper and lower panels show *SIRH11/ZCCHC16* and *ACTB* agarose gel electrophoresis profiles, respectively. HEK293T genome was amplified as a control for PCR. Human total RNA was purchased from Clontech (Human total RNA Master Panel II, #636643). The cDNA was made from total RNA (1 μg) using Revertra Ace qPCR RT Master Mix (TOYOBO, Japan). 10 ng of cDNA was used for RT-PCR analysis. The PCR reaction was performed using Ex*Taq*HS polymerase (TaKaRa, Japan) with following condition: 96°C, 1 min; 30 cycles (for *ACTB*) or 35 cycles (for *SIRH11/ZCCHC16*) of 98°C, 10 s; 60°C, 30 s; 72°C, 30 s; final extension 72°C, 3 min. The following primer sequences were used: hACTB_F: 5′-AAGTGTGACGTGGACATCCG-3′ and hACTB_R: 5′-GATCCACATCTGCTGGAAGG-3′; hSIRH11_F: 5′-GGTGACCCTGCCAATTGCTC-3′ and hSIRH11_R: 5′-AGGTACTCTTGTCAGGCCCAG-3′.
