# Supplementary material for: An LTR Retrotransposon-Derived Gene Displays Lineage-Specific Structural and Putative Species-Specific Functional Variations in Eutherians
Source: Front Chem. 2016 Jun 23;4:26. doi: 10.3389/fchem.2016.00026 (PMC4917564; doi:10.3389/fchem.2016.00026)
Supplement: Figure S1 — Alignment of amino acid sequences of SIRH11/ZCCHC16 from 83 eutherian mammals (related to Figure 1). [file DataSheet1.pdf]

```
human                                      MEKCTKSSSTMQVEPS----FLQAENLILRLQMQHPTTENTAKRGQVMPALATTVMPVPY        56
chimpanzee                                 MEKCTKSSPTMQVEPS----FLQAENLILRPQMQHPTTENTAKRGQVMPALATTVMPVPY        56
pygmy_chimpanzee                           MEKCTKSSPTMQVEPS----FLQAENLILRPQMQHPTTENTAKRGQVMPALATTVMPVPY        56
western_lowland_gorilla                    --------------------------------MQHPTTENTAKRGQVMPALATTVMPVPY        28
Sumatran_orangutan                         ----MKSPPTMQVEPS----FLQAENLILRPQMQHPTTENTAKRGQVMPALATTVMPVPY        52
northern_white-cheeked_gibbon_a            MEKCTKSPPTMQVEPS----FLQAENLILRPQMQHPTTENTAKRGQVMPALATTVMPVPY        56
northern_white-cheeked_gibbon_b            ------------------------------------------------------------        0
sooty_mangabey                             MEKCTKSPPTLQVEPS----FLQGENLILRPQMQHPTTENTAKRGQVMPALATTVMPVLN        56
green_monkey                               MEKCTKSPPTLQVEPS----FLQAENLILRPQMQHPTMENTAKRGQVMPALATTVMPVPN        56
crab-eating_macaque                        MEKCTKSPPTLQVEPS----FLQGENLILRPQMQHSTTENTAKRGQVMPALATTVMPVPN        56
rhesus_monkey                              MEKCTKSPPTLQVEPS----FLQGENLILRPQMQHSTTENTAKRGQVMPAMATTVMPVPN        56
pig-tailed_macaque                         MEKCTKSPPTLQVEPS----FLQGENLILRPQMQHPTTENTAKRGQVMPALATTVMPVPN        56
drill                                      MEKCTKSPPTLQVEPS----FLQGENLILRPQMQHPTTENTAERGQVMPALATTVMPVPN        56
olive_baboon                               MEKCTKSPPTLQVEPS----FLQGENLILRPQMQHPTTENTAKRGQVMPALATTVMPVPN        56
Colobus_angolensis_palliatus               MEKCTKSPPTLQVEPS----FLQAENLILRPQMQHLTTENTAKRAQVMPALATTVMPVPN        56
golden_snub-nosed_monkey                   MEKCTKSPPTLQVEPS----FLQAENLILQPQMQHPTTENTAKRAQVMPALATTVMPVLN        56
Mas_night_monkey                           ------------------------------------------------------------        0
common marmoset                            ------------------------------------------------------------        0
Bolivian_squirrel_monkey                   ------------------------------------------------------------        0
Philippine_tarsier                         MDKCTESPTTLQVESSDS-AVTNAENPILQSQMQHPIRENSALRGQVMPALAIPVMPVPF        59
gray_mouse_lemur                           MEKYMESPPTLQAEPS----FPQAENLTLQPQMQHPTKENSALKDQGMPALATPAMTVPC        56
Coquerels_sifaka                           MEKYMESPPTLQVEPS----FPQAENLTLQPQMQHPTNDNPALRGQDMPALATPVMTVPC        56
small-eared_galago                         MEKYTESPPTLQVEPS----FPQAENLTLRPQVQHSTKESPALRGQVMPALATPGINVPC        56
Damara_mole-rat                            ------------------------------------------------------------        0
naked_mole-rat                             ------------------------------------------------------------        0
domestic_guinea_pig                        ------------------------------------------------------------        0
long-tailed_chinchilla                     ------------------------------------------------------------        0
degu                                       ------------------------------------------------------------        0
lesser_Egyptian_jerboa                     MEKCTESPPTVQVEGS----FLGGDNLTLQPQMQDLTKDNTALRRQGMPVLNTPMMPVSY        56
Ords_kangaroo_rat                          MEKCAESPPTLQVEPS----FPWGDNVMMQRHMQHLSEDNPTLKSQVMPPVTTAVTSIPY        56
prairie_vole                               MEKCTESPPTLKAETS----LLKGDNLIVPSQIEHPTDDNPTLREQVVPVPNIPVMSVPY        56
Chinese_hamster                            MEKCTKSPPTLKPETS----FLRGDSLILQPQMQHPTDDNPTLRGQVVPVLNTPVMSIPY        56
golden_hamster                             MEKCTESPPTLKVETS----FSRGDNLVLQPQMQHPTDDNPTLRGQVVPVLNTPMMSTPY        56
prairie_deer_mouse                         MEKCTESPPTLKAGPS----FLRRDNLILQPQMQHPTDDNPTLRGQVVPVPDTPVMSLPY        56
house_mouse                                MEKCTESLPNLNAETS----FLRGGNLILQPQVQHPTDDKPPITGQVVPALNTPVMSGPY        56
Norway_rat                                 MEKCTESPPNLNAETS----FLRGGNLIQQPQVQHPADEKPPLTGQVVPPLNSPVMSGPY        56
Upper_Galilee_mountains_blind_mole_rat     --------------------------MIVQSQMQHLTDDNPALRNHVIPVSNTPVMSVSY        34
thirteen-lined_ground_squirrel             MEKCTESPPTLQVEPS----FLQGENLILQPQMQHLTEENPALRGQLMPPLTTPVMSVPY        56
rabbit                                     MEECTESSSTLQVEPS----FPQAENLNLQSQMQHLAKENLALRGQGTPTLASAMMPVPC        56
American_pika                              MDKYTESAPTLQVEPS----FLQGENLNQQPQIQCLTKENLDFGDQVMPTLATPMMPVPC        56
Chinese_tree_shrew                         MEKCTKSPSTLQGESS----FLQAENLILQPQMPHLSKENPTLKGQITPALATSVMSIPC        56
Sunda_flying_lemur                         MERCTESPPTLQVEPS----FIQSEKLILQPQMQHLTKEEPALRGQVMPASGTPVIPVPC        56
dog                                        MEKYTESPSTLQVEHS----SLQVENLILPPQIQHLTEENLDLRGQVMPTLTTPVMSVTC        56
domestic_ferret                            MEKYTESPPTLQVEHS----SLQTENLILQPQIQRLNEEGSDLRGQVMPTLATVMMSVPR        56
Pacific_walrus                             MEKYTDSPPTLQVEHS----SLQTENLILQPQIQHLTEESPDPRGQVMPTLATPMMSVPC        56
Weddell_seal                               MEKYTDSPPTLQVEHS----SLQTENLILQPQIQHLTEENPDPRGQVMPTLATPMVSVPC        56
giant_panda                                MEKYTGSPPTLQVEHS----SLQTENLILQPQIQHLTEESPHLRGQVMPTLATPMMSVPR        56
polar_bear                                 MEKYTRSSPTLQVEHS----SLQTENLILQPQIQHLAEESPDLRGQVMPTLATPMMSVPR        56
domestic_cat                               MEKYTETPPSLQVEHS----SLQEENLFLQPQIQHLTEENPELRGQIMPALATPLMSVPC        56
Amur_tiger                                 MEKYTETPPTLQVEHS----SLQEENLFLQPQIQHLTEENPELRGQIMPALATPLMSVPC        56
Balaenoptera_acutorostrata_scammoni        ------------------------------------------------------------        0
killer_whale                               ------------------------------------------------------------        0
bottlenosed_dolphin                        ------------------------------------------------------------        0
Yangtze_River_dolphin                      ------------------------------------------------------------        0
sperm_whale                                ------------------------------------------------------------        0
chiru                                      ------------------------------------------------------------        0
Bison_bison_bison                          ------------------------------------------------------------        0
cattle                                     ------------------------------------------------------------        0
wild_yak                                   ------------------------------------------------------------        0
water_buffalo                              ------------------------------------------------------------        0
goat                                       ------------------------------------------------------------        0
sheep                                      ------------------------------------------------------------        0
mouflon                                    ------------------------------------------------------------        0
pig                                        MENYIDSPPTLQVEHS----SLQAENLILQPQMQHVTEENPALRGQVMSALTPPMMPVPC        56
Bactrian_camel                             MENYTESPPTLQVEHS----SLWAENLNPQPHMWHLTEKNPALRDQLMPALATPVMPVPC        56
Arabian_camel                              MENYTESPPTLQVEHS----SLWAENLNPQPHMWHLTEKNPALRDQLMPALATPVMPVPC        56
Wild_Bactrian_camel                        MENYTESPPTLQVEHS----SLWAENLNPQPHMWHLTEKNPALRDQLMPALATPVMPVPC        56
alpaca                                     MENYTESPPTLQVEHS----SLWAENLNPQPHMWHLTEKNPALRDQLMPALATPVMPVPC        56
black_flying_fox                           MEKYTESSPTMQVEHS----FFQVENPILEPQVQHLTEDNATLREQVMSALATPMMPISC        56
large_flying_fox                           MEKYTESSPTVQVEHS----FFQVENPILEPQVQHLTEDNATLREQVMSALATPMMPIFC        56
big_brown_bat                              MEKHTESSPRLQVEHS----SLWVENLILQPQVQHLTEENTTLRDQVMPALATPMMPLLC        56
little_brown_bat                           MEKHTESSPTLQVEHS----SLWAENLILQPQVQHLTEENTTLRDQVMPALATPMMPLLC        56
Brandts_bat                                MEKHTESSPTLQVEHS----SLWAENLILQPQVQHLTEENTTLRDQVMPALATPMMPLLC        56
Myotis_davidii                             MEKHTESSPTSQVEHS----SLWAENLILQPQVQHLTEENTTLKDKVMPALATPMMPLLY        56
European_shrew                             MEKCTQSPPILKVEHL----SLQAENMILESQWDHPTKENPDLRSQAISAVDITEMSESD        56
star-nosed_mole                            MEKHPESPPALQVEQP----SLQEENRTLPWQVQHLPEENHVLKGQVKPGMATSVMPVPF        56
horse                                      MEKYTESPPALQVEHA----SLQAENLILRPQMQYLTEENPAMRGQVMPSLATPMMPVSC        56
Przewalskis_horse                          MEKYTESPPALQVEHA----SLQAENLILRPQMQYLTEENPAMRGQVMPSLATPMMPVSC        56
southern_white_rhinoceros                  MEKYTESPPTLQVEHS----SLQAENLIPRPQKQHLTEENPAMKDQVMPALSTLMMPISC        56
Cape_golden_mole                           MEKLTDSPPILKIEHP----SLWADNLILEPQVQHLTEENTALRGQVMLNLATTMLPVPC        56
Cape_elephant_shrew                        MEKTRESSPALQGGESDSGANLWAENMILGPPMQPLTEGNTTLRSQVMPSLATPMMPIPF        60
African_savanna_elephant                   MENFTESPPSLQVEHS----SLWADNLILEPQVHHLTEDNTALRGQVMPTLATPMMPVSS        56
Florida_manatee                            MEKFTESPPTLQVEHS----SLWADDLILEPQVQHLTDDSTALRGQVMPTLATPMMPVSC        56
small_Madagascar_hedgehog                  MEKLPASPPTLQVEHS----SLLTENQIPESQGQTLTEENIALKGPVIPALATTMVPAPC        56
Orycteropus_afer_afer                      MEKHTDSPPTLQGEPS----SLWAENLILEPQEQHLTEDNISLKDQVMPTPATSMMPVPC        56
```

Fig. S1

```
human                                      SLEHLTQFHG-DPANCSEFLTQVTTYLTALQISNPANDAQIKLFFDYLSQQLESCGIISG     115
chimpanzee                                 SLEHLTQFHG-DPANCSEFLTQVTTYLTALQISNPANDAQIKLFFDYLSQQLESCGIISG     115
pygmy_chimpanzee                           SLEHLTQFHG-DPANCSEFLTQVTTYLTALQISNTANDAQIKLFFDYLSQQLESCGIISG     115
western_lowland_gorilla                    SLEHLTQFHG-DPANCSEFLTQVTTYLTALQISNPANDAQIKLFFDYLSQQLESCGIISG     87
Sumatran_orangutan                         SFEHLTQFHG-DPANCSEFLTQVTTYLTALQISNPANDAQIKLFFDYLSQQLESCEIISG     111
northern_white-cheeked_gibbon_a            SLEHLTQFHG-DPANCSEFLTQVTTYLTALQISNPANDAQINVFFDYLSQQLESCGNISG     115
northern_white-cheeked_gibbon_b            ------------------------------------------------------------     0
sooty_mangabey                             SLEHLTQFHG-DPANHSEFLTQVTTYLTALQISNPANEAQIKLFFDYLSQQLESCGIISG     115
green_monkey                               SLEHLTQFHG-DPANRSEFLTQVTTYLTALQISNPANDAQIKLFFDYLSQQLENCGIISG     115
crab-eating_macaque                        SLEHLTQFHG-DPANRSEFLTQVTTYLTSLQISNPANDAQIKLFFDYLSQQLESCGIISG     115
rhesus_monkey                              SLEHLTQFHG-DPANRSEFLTQVTTYLTSLQISNPANDAQIKLFFDYLSQQLESCGIISG     115
pig-tailed_macaque                         SLEHLTQFHG-DPANRSEFLTQVTTYLTSLQISNPANDAQIKLFFDYLSQQLESCGIISG     115
drill                                      SLEHLTQFHG-DPANRSEFLTQVTTYLTALQISNPANEAQIKLFFDYLSQQLESCGIISG     115
olive_baboon                               SLEHLTQFHG-DPANRSEFLTQVTTYLTALQISNPANDAQIKLFFDYLSQQLESCGIISG     115
Colobus_angolensis_palliatus               SLEHLTQFHG-DPASRTEFLTQVTTYLTALQISNPANDAQIKLFFDYLSQQLESCGIISG     115
golden_snub-nosed_monkey                   SLEHLTQFHG-DPANRSEFLTQVTTYLTALQISNPANDAQIKLFFDYLSQQLESCGIISG     115
Mas_night_monkey                           ------------------------------------------------------------     0
common marmoset                            ----------------------MTTYLTALQISNPANDAQINLFIDYLFQKLESCGIISG     38
Bolivian_squirrel_monkey                   ------------------------------------------------------------     0
Philippine_tarsier                         SLEHLTHFHG-DPASLSGFLAQVTTYMTALQISNPEHDAHVKLFFDYLSQQLESCGVVSG     118
gray_mouse_lemur                           SFEHLTKIHD-DPASLAGFLAQVTTYLTDLQLSNPSDDARVKLFFGYLSQQLGSCGAISR     115
Coquerels_sifaka                           SLEHLTQIRG-DPDSLSGFLAQVTTYLTALQISDPSDDAQVKFFFDYLSQQLGSCGAISG     115
small-eared_galago                         SLEHLTQFHG-DPASLSGFLAQVTTYLTALQISNHSDDAQAKLFFDYLSQQLGSCGVMSG     115
Damara_mole-rat                            ------------------------------------------------------------     0
naked_mole-rat                             ------------------------------------------------------------     0
domestic_guinea_pig                        ------------------------------------------------------------     0
long-tailed_chinchilla                     ---------------------------------------------------METFGIYSE     9
degu                                       ---------------------------------------------------MENFGINCE     9
lesser_Egyptian_jerboa                     VLEHLPRLHG-DPSSLSGFLAQVTTYMTALKISNPADDAQVKHFFEYLSQQMQKHGVISE     115
Ords_kangaroo_rat                          SLEHHTEFHS-ESSNLSGFLAQVTTYLSPLKCPNPTEDAEVKHFCDVPIHQVQRCGIISG     115
prairie_vole                               SGNHLPQFHG-KPTSATGFLAQVTTYLTALKISNPADDARVKHFFDCLSQQMQSCDVVSE     115
Chinese_hamster                            SGDNLPQFHG-EPTSVTGFLAQGTTYLTAPEISNPADDTRVKHFFDYLSQQVQSCDIVSE     115
golden_hamster                             SSGNLPQFHG-ETTSVTGFLAQGTTYLTALEISNPADDARVKHFFDYLSQQMQSCDIVSE     115
prairie_deer_mouse                         SGDHLPQFHD-ESTSVTGFLARVTTYLTALKISNPADDARVKHFFDYLSQQMQSCDIVSE     115
house_mouse                                SGDHIPQFHG-NPASVKGFFAQVTTYLRALDISNPADNARVKHFFDYLSQQMQNCDVLSE     115
Norway_rat                                 SGDHLPQFHG-NPASVRGFFAQVTAYLTTLDISNPADDARVKHFFDYLSQQMQNCDVLSE     115
Upper_Galilee_mountains_blind_mole_rat     SLEHLFQVHD-DPTSVSEFRAQVTTYLTSLKISNPADDAQVKFSFEYLSQQMQISDVKTG     93
thirteen-lined_ground_squirrel             SLENLTQFHG-DPASVSGFLAQVTTYLTAFKIPNSVNGTQNKLVFDYLSQQMESCGVTSG     115
rabbit                                     SLEHFDQFHD-DPASLSGFLAQVTTYLTALKRNNPTDGDKVKLFFDYISQQMKSYRVTSG     115
American_pika                              LLEHFAQFHG-DPVSLSRFLAQITTYLNALKKNNPADDDQVKLIFDYISQQIENCRVTSG     115
Chinese_tree_shrew                         SLEHLTQFHD-DPARLSELLAQMTTYLTDLQIPNPTDNGQVKHFFDYLSQEMENCGVISG     115
Sunda_flying_lemur                         SLEHFTQFHG-DPASFSEFLAQVTTYLTALQITNPENDVQVKLFFDYLSQQMESYGVISG     115
dog                                        LLEHLSQFHS-DPASISGFPARL----TTLKFPNPADDVQIKFFFDYLSQQMEGCGVISG     111
domestic_ferret                            SLEHLSQFHH-DPANLSIFLAQL----TTLKFPSPADDIQVKFFFDYLSQQMESCGVIFG     111
Pacific_walrus                             SLEHLSQFHH-DPANLSRFLAQL----ATLKFPNPTDDVQVKFFFDYLSQQMESCGIISG     111
Weddell_seal                               SLEHLSQFHH-DPANLSRFLAQL----TTLKFPNPADDVQVKFFFDYLSQQMESCGIISG     111
giant_panda                                SLEHLSQFHR-DPANLSKFLAQL----TTFKFPNPADDVQVKFFFDYLSQQMEICGVVSG     111
polar_bear                                 SLEHLSQFHH-DPANLSRFLVQL----TTFKFPNPADDVQVKFFFDYLSQQMEICGVVSG     111
domestic_cat                               SLEHLSQFHG-DPDNL------L----TTVKIPNPADDVQVKFFLNYLAQQMESCGVISG     105
Amur_tiger                                 SLEHLSQFHG-DPDNL------L----TTVKIPNPADDVQVKFFLNYLAQQMESCGVISG     105
Balaenoptera_acutorostrata_scammoni        ------------------------------------------------------------     0
killer_whale                               ------------------------------------------------------------     0
bottlenosed_dolphin                        ------------------------------------------------------------     0
Yangtze_River_dolphin                      ------------------------------------------------------------     0
sperm_whale                                ------------------------------------------------------------     0
chiru                                      ------------------------------------------------------------     0
Bison_bison_bison                          ------------------------------------------------------------     0
cattle                                     ------------------------------------------------------------     0
wild_yak                                   ------------------------------------------------------------     0
water_buffalo                              ------------------------------------------------------------     0
goat                                       ------------------------------------------------------------     0
sheep                                      ------------------------------------------------------------     0
mouflon                                    ------------------------------------------------------------     0
pig                                        SLEHLSQFHG-DPTNISEFLAQMTTYLTTLKIPNPEDNAHVKFIFDHLSQQMESCGVISG     115
Bactrian_camel                             SLEHLSQSHD-DPASLSGLLAQVTTSSTTFKITNPADDAHVKFSFDYLPQQMEVCGVVSG     115
Arabian_camel                              SLEHLSQSHD-DPASLSGLLAQVTTSSTTFKITNPADDAHVKFSFDYLPQQMEVCGVVSG     115
Wild_Bactrian_camel                        SLEHLSQSHD-DPASLSGLLAQVTTSSTTFKITNPADDAHVKFSFHYLPQQMEVCGVVSG     115
alpaca                                     SLEHLSQSHD-DPASLSGLLAQVTTSSTTFKITNPADDAHVKFSFDYLPQQMEVCGVVSG     115
black_flying_fox                           SLEHLSQFYD-DTANISGFLAQVTTYLTILKIPNPADDAQVKFFFDYLSQQMESCGIIST     115
large_flying_fox                           SLEHLSQFYD-DTANVSGFLAQVTTYLTILKIPNPADDAQVKFFFDYLSQQMESCGIIST     115
big_brown_bat                              SLKNLSQFRG-DPANISGFLAQVTNYLTTLKNTNPIHDDQVKFFFDHLSQQMESCGMISR     115
little_brown_bat                           SLKNLSQFHD-DPANLSGFLAQVTNYLTTLKITNPIHDAQVKLFFDYLSQQMESCGIISR     115
Brandts_bat                                SLKNLSQFRD-DPANLSGFLAQVTNYLTTLKITNPIHDAQVKLFFDYLSQQMESCGIISR     115
Myotis_davidii                             SLKNLSQFPD-DPANLSGFLAQVTNYLTTLKITNPIHDAQVKLFFDYLSQQMESCGMISR     115
European_shrew                             SFGYPSQVPGDDSANISKFLAQMTTCLSTTKLPKSPNDDQAKVCFDHKSQQMESCQVISG     116
star-nosed_mole                            SSEHPSKFHG-VPANLSGFLAEVTTYLTTPMVSNTADDTQVKLFFDCLSQQMEKCGVMSG     115
horse                                      SLEHLSQFRD-DPASLSGFLAQVTTYLTALKIPNPADDAQVKFFFDYLYQQMKSCGIISG     115
Przewalskis_horse                          SLEHLSQFRD-DPASLSGFLAQVTTYLTALKIPNPADDAQVKFFFDYLYQQMKSCGIISG     115
southern_white_rhinoceros                  SPEHLSQFHG-DPANLARFLAQVTTYLTTLKIPNPADDAQIKFFFDYLYQQMESCGVISG     115
Cape_golden_mole                           SLEHLNQFHV-DPANLSGFLAQATTYLASLN--YPADNAKAKFFIDYILQQVKRSGVLHG     113
Cape_elephant_shrew                        SPEHLSKFHV-NPASLSGFLAQATAYFTALKNSNTADNAQVMSFLEYISRQMERYGTLPG     119
African_savanna_elephant                   SLEHLSQFHV-DPANLSGFLAQATTYLTTLKTPNPADDVQVKFFFDYISRQVERCRILPG     115
Florida_manatee                            SLEHPSQFHV-DPANLSGFLAQATTYLTALKTPNPADDVQVKFFFDYISQQVERCRILPG     115
small_Madagascar_hedgehog                  LFEHLNQFHV-DPDNPSELLAQVTTYLTALKPSNPAENTQIKCFIDYISQQAKKCGVLPG     115
Orycteropus_afer_afer                      SPEHLGQFDA-DPASISGFLAQATTDLTALKTLNPVDNAQVKFFFDYISQQVERCGVLAG     115
```

Fig. S1 (continued)

human	PDKSTLLKQYENLILEFQQSFGKPTKQEINPLMNAKFDKGDNSSQQDPATFHLLAQNLIC	175
chimpanzee	PDKSTLLKQYENLILEFQQSVGKPTKQEINPLMNAKFDKGDNSSQQDPATFHLLAQNLIC	175
pygmy_chimpanzee	PDKSTLLKQYENLILEFQQSFGKPTKQEINPLMNAKFDKGDNSSQQDPATFHLLAQNLIC	175
western_lowland_gorilla	PDKSTLLKQYENFILEFQQSFGKPTKQEINPLMNAKFDKGDNCSQQDPATFHLLAQNLIC	147
Sumatran_orangutan	PDKSTLLKQYENFILEFQQSFGKPTKQEMNPLMNAKFDKGDNSSQQDPTTFHLLAQNLIC	171
northern_white-cheeked_gibbon_a	PDKSTLLKQYENFILEFQQSFGKPTKQEMNPLMNAKFDKGDNSSQQDPTTFHLLAQNLIC	175
northern_white-cheeked_gibbon_b	--	0
sooty_mangabey	PDKSTLLKQYENCILEFQQSFGKPTKQEMNLLMNAKFDKGDNSSQQDPTTFQLLAQNLIC	175
green_monkey	SDKSTLLKQYENCILEFQQSFGKPTKQEMNPLMNAKFEKGDNSSQLDPTTFQLLAQNLIC	175
crab-eating_macaque	PDKSTLLKQCENCILEFQQSFGKPTKQEMNLLMNAKFDKGDNSSQQDPTTFQLLAQNLIC	175
rhesus_monkey	PDKSTLLKQCENCILEFQQSFGKPTKQEMNLLMNAKFDKGDNSSQQDPTTFQLLAQNLIC	175
pig-tailed_macaque	PDKSTLLKQYENCILEFQQSFGKPTKQEMNLLMNAKFDKGDNSSQQDPTTFQLLAQNLIC	175
drill	PDKSTLLKQYENCILEFQQSFGKPTKQEMNLLMNAKFDKGDNSSQQDPTTFQLLAQNLIC	175
olive_baboon	PDKSTLLKQYENCILEFQQSFGKPTKQEMNLLMNAKLDKGDNSSQQDPTTFQLLAQNLIC	175
Colobus_angolensis_palliatus	PDKSTLLKQYENCILEFQQSFGKPTKQEMNLLMNAKFDKGDNSSQQDPTTFQLLAQNLIR	175
golden_snub-nosed_monkey	PDKSTLLKQYENCVLEFQQSFGKPTKQEMNLLMNAKFDKGDNSSQQDPTTFQLLAQNLIC	175
Mas_night_monkey	----------------------------MNPLMNAKVDKGDNSSQQDPATFQLFAQNLIC	32
common marmoset	PDKSTLLKQYENFILEFQQLLGEPTKQEMNPLMNAKVDKGDDSSQQDPATFQLLAQNMIC	98
Bolivian_squirrel_monkey	----------------------------MNPLMNDKVDKGDNSSQQDPATFQLLAQNLIC	32
Philippine_tarsier	PDQSTLLKQCENFVLEFQQSFGEPTKQKMSPMMNKTVDKGDNS-QQDASTFKLFAQNLSC	177
gray_mouse_lemur	PDQSTLLKQYENFVLEFQQSFGESKKQEMNPLMNTKVDKGDNSSQQDITTFQLFAQNLSC	175
Coquerels_sifaka	PDQSTLLKQYENFVLEFQQSFGEPTKQETNPLMNTKVDKGDNSSQQDITTFQLFAENLSC	175
small-eared_galago	PDQGTLLKQYENFVLEFQRSFGDSTKQEMNPLMNTKVDKGNNSFQQDTTTFQLFAQNLSS	175
Damara_mole-rat	----------------------------MNHLMDAKVDRGDSTSQQDVTTFQFFSQNLSS	32
naked_mole-rat	----------------------------MNPLMNDKVDKGDNTSQQDVTTFQFLSQNLTC	32
domestic_guinea_pig	----------------------------MNPLMNSKVEKGDNTSQQDVTTFQFFSQNLSC	32
long-tailed_chinchilla	YNQ-----QNENCVLDFQPSSGEPKKQEMNPLMNPKVDKGDSTSHQHVTTFQFSSQSLSY	64
degu	YDQCAPLKENENFALDFQQSSRDPIEQEMNLLMNAKVDKVDSGFQQNITTFQFLSQSLSC	69
lesser_Egyptian_jerboa	SNQNILLEQCENFVLEFQQSFGEPMQQEINPLMNVKVNKGDKISHQDAKPSQILAQNLSY	175
Ords_kangaroo_rat	SDHSAVLQQYENFVLEFQKSFDEPMQQEMNPLIFADADKENNSSQQDAGTFQGFTLNMNC	175
prairie_vole	SNQSNLLKQYENLLLELQQSFGEPMKQEITLPMNVKVDSRDNN-QQDATAFQVLPQNLSY	174
Chinese_hamster	SNQSHLLKQYENFILELKQSIGEPMKEEISPLMNVKNDSRDNS-QQDAPTFQGLAQNMSY	174
golden_hamster	SNQSHILKQYENCILELKQSFGEPVKQEITPPMNVTVDSKDNS-QQDATTFQGLPQNLNY	174
prairie_deer_mouse	SNQSNLLKQYENFVLELQQSCGEPMKQEIIPPINVKVDSRDNS-QQDATTFEGFAQNLSD	174
house_mouse	STQNNLLKQYENFVLELQQSFGEPMTQETTPPMNVTVDKSNIS-PQDATNFQLHAPNLSY	174
Norway_rat	SSQRNLLKQYENFVLEFQKSFGEPMIQETTPPMNAMVDKGDIS-PQDATTSQVHAPNLSY	174
Upper_Galilee_mountains_blind_mole_rat	SDQISLLKLYENFILKFQQSFGESMKQEMNPLTNVKVDKGKNSSQQDSTNFQILPQNLSY	153
thirteen-lined_ground_squirrel	SDQSTILKQHESFVHDFQQSFDEPMKQELNPLMNTKVDKGDNPS-----------QNVTC	164
rabbit	PNENTMLKQYENFVLEFQRSFGEATKQEMNPLINAQVVKGNTSSDQDTTILQLLTPHLSC	175
American_pika	PNQNAMLKQYENFVFEFQQSFGKPKKQELNPLLNAQVVKGNACSHQATTTPHLLASNPSS	175
Chinese_tree_shrew	SDKRTLLKQYENLVLEFQQSFGEPIKQETNPLLNAKVNKGYNSSQQDSTTFWLLAQNLNC	175
Sunda_flying_lemur	ADQSTMLKQYENFVLEFQKQFCEPTKQEMNSLVNAKVEKGDNSSQQDTTAFRLLAQNLSC	175
dog	SDQNTLLKQYEKFVLEFQQSFGEPIKQEIDPLN----KEDNSSSQQDASTFQLLAQNLIC	167
domestic_ferret	PDQSSQQKQYENFVFAFQQSLGVPTKQEIDTLN----KEDNS-SQKDASTFEHLAQNMSY	166
Pacific_walrus	PDQSTLLKQYENFVLEFQQSFGEPTKQEIDPLN----KKDNSSPQQHASTFQLLAQNLSC	167
Weddell_seal	PDQSTLLKQYENFVLEFQQSFGEPTKQEIDLLN----KKDNSSSQQDASTFQLLAQNLSC	167
giant_panda	PDQSTLLKQYENFVLEFQQSFGGPTKQEIDPLN----KEVNSFSQQDASTLQLLAQNLSC	167
polar_bear	PDQSTLLKQYENFVLECQQSFGGPTKQEIDPLN----KEVNSFSQQEASTFQLLAQNLSC	167
domestic_cat	PNQSTLLKQYEKFVFEFQQSFGEPTKQETDPMN----KEDNS-SQQNASTFNLLAQNLSC	160
Amur_tiger	PNQSTLLKQYEKFVFEFQQSFGEPTKQETDPMN----KEDNS-SQQNASTFNLLAQNLSC	160
Balaenoptera_acutorostrata_scammoni	----------------------------MNPLVNAKVDKRDNFSQQDATTFHLLAQNLRY	32
killer_whale	----------------------------MNPLMNAKVDKRDNFSQQDATTFQLLAQNLRY	32
bottlenosed_dolphin	----------------------------MNPLMNAKVDKRDNFSQQDATTFQLLAQNLRY	32
Yangtze_River_dolphin	----------------------------MNPLVNAKVDKRDDFSQQDATTFQLLAQNLRY	32
sperm_whale	----------------------------MNPLVNAKVDKRDNFSQQDATTFQLLAQNQRY	32
chiru	----------------------------MNRLVNTKVDKEDNFSQQNASMFHLLAQNLRY	32
Bison_bison_bison	----------------------------MNRLVNTKVDKEDNFSQQNASTFHLLAQNLRY	32
cattle	----------------------------MNRLVNTKVDKEDNFSQQNASTFHLLAQNLRY	32
wild_yak	----------------------------MNRLVNTKVDKEDNFSQQNASTFHLLPQNLRY	32
water_buffalo	----------------------------MNHLVNTKIDKEDNFSQQNASTFHLLAQNLRY	32
goat	----------------------------MNHLVNTKVDKEDNFSQQNASTFHLLAQNLRY	32
sheep	----------------------------MNHLVNTKVDKEDNFSQQNASTFHLLAQNLRY	32
mouflon	----------------------------MNHLVNTKVDKEDNFSQQNASTFHLLAQNLRY	32
pig	PDQSALLKQHENFVLEFQQSFGELTKQ-EMKSLVTKVDKGDKFSQQDTTTFQLLAQNLRY	174
Bactrian_camel	PDESALLEQYENFVPDFQQSFGEPTEQEMNPLVNAKVDKENSFSQQDATTFQLLAQNLRY	175
Arabian_camel	PDESALLKQYENFVPDFQQSFGEPTEQEMNPLVNAKVDKENSFSQQDATTFQLLAQNLRY	175
Wild_Bactrian_camel	PDESALLEQYENFVPDFQQSFGEPTEQEMNPLVNAKVDKENSFSQQDATTFQLLAQNLRY	175
alpaca	PDESALLKQYENFVPDFQQSFGEPTKQEMNPLVNAKVDKENSFSQQDATTFQLLAQNLRY	175
black_flying_fox	PDQNALLKQYENFVLEFQQSFDESKKQEMSSLVNDKIDKGDKFSKQDATTFQLLGQNLNC	175
large_flying_fox	PDQNALLKQYENFVLEFQQSFDESKKQEMSSLVNDKIDKGDKFSKQDATTFQLLGQNLNC	175
big_brown_bat	SEQSALLKQYENFVLEFQQSFGESKRQEMNPLVNAKVDEEDNSSQQDATTFQPLAQNLSC	175
little_brown_bat	SEQSALLKQYENFVLEFQRSFGESKRQEMSPLVNAKVDEGDNSSQQDATTFQPLAQNLSC	175
Brandts_bat	SEQSALLKQYENFVLEFQRSFGESKRQEMSPLVNAKVDEGDNSSQQDATTFQPLAQNLSC	175
Myotis_davidii	SEQSALLKQYENFVLEFQRSFGESKRQAMSPLMNAKVDEGDNSSQQDATTFQPLAQNLSC	175
European_shrew	PYQSTLMKQNEKYILEF---LCEPTNREVNSLENIKVDKGDNPQ-QNAIIF----KHLSS	168
star-nosed_mole	IDQNTLMRQYEDIVLEFQPSVSKPVKQEIHPLVNAKTDKGDNS-QQDVISFQLPARNLSC	174
horse	PDKRTLLKQYENFVLEFQQSFGELTKQKRN-----NLDKGDNS-QQDATTFQILAQNLSY	169
Przewalskis_horse	PDKRTLLKQYENFVLEFQQSFGELTKQKRN-----NLDKGDNS-QQDATTFQILAQNLSY	169
southern_white_rhinoceros	PDQSTLLKQYENFVLEFQQSFGELTKEKMN-----HLDKGDNSSQQDATTFQLLAQNLSN	170
Cape_golden_mole	SNQRALLKQYENFVFEFQQLFGEPTKQVMGPLETAKVDKGDDLFQEYATAFQLLAQNMDC	173
Cape_elephant_shrew	SEQNTLMKQYEKFLFEVQQSFGESTKQEAKPLVNAHAGKRNTTSHHDATTFQLLPQNSNS	179
African_savanna_elephant	SDQNTLLKQYENFVFEFQQLFGEPTKQEMNPLVNAKVDKGDDSFQQYATTFQHLAQNMNC	175
Florida_manatee	SDQSTLLKQYENFVSEFQQSFGEPTKQEMNPLVNAKLDKGNDSFQQYATTFQLLAENISC	175
small_Madagascar_hedgehog	SDQSILLKQYESLVFEFQGAFGERTEQEMNPLLNAEIDKEDDSFQQ------LLAQNMNC	169
Orycteropus_afer_afer	SDQSTLLKQYENFVFEFQQSFGKPTKQEMNSLANAKVDKGDDSFPKCA-TLQLLAQKMNC	174

Fig. S1 (continued)

human	NET--NQSGQFEKALADPNQDEESVTDMMDNLPDLITQCIQLDKKHSDRPELLQS-ETQL	232
chimpanzee	NET--NQSGQFKKALADPNQDEESVTDMMDNLPDLITQCIQLDKKHSDRPELLQS-ETQL	232
pygmy_chimpanzee	NET--NQSGQFKKALADPNQDEESVTDMMDNLPDLITQCIQLDKKHSDRPELLQS-ETQL	232
western_lowland_gorilla	IET--NQSGQFEKALADPNQDEESVTDMMDNLPDLITQCIQLDKKHSDRPELLQS-ETQL	204
Sumatran_orangutan	NET--NQSGQFEKALADPNQDEESVTDMMDNLPDLITQCIQLDKKHSDRPELLQS-ETQL	228
northern_white-cheeked_gibbon_a	NET--KVVSSKRH*---	186
northern_white-cheeked_gibbon_b	---------------------------MMDNLPDLITQCIQLDKKHSDRPELLQS-ETQL	32
sooty_mangabey	NET--SQNGQFEKAVDDLNQDEESVTDMMDNLPDLITQCIQLDKKHSDRPELLQS-ETQL	232
green_monkey	NET--NQNGQFKKTVDDLNQDEESVTDTMDNLPDLITQCIQLDKKQSDRPELLQS-ETQL	232
crab-eating_macaque	NET--SQNGQFEKAVDDLNQDEESVTDMMDNLPDLITQCIQLDKKHSDRPELLQS-ETQL	232
rhesus_monkey	NET--SQNGQFEKAVDDLNQDEESVTDMMDNLPDLITQCIQLDKKHSDRPELLQS-ETQL	232
pig-tailed_macaque	NET--SQNGQFEKAVDDLNQDEESVTDMMDNLPDLITQCIQLDKKHSDRPELLQS-ETQL	232
drill	NET--SQNGQFEKAVDDLNQDEESVTDMMDNLPDLITQCIQLDKKHSDRPELLQS-ETQL	232
olive_baboon	NET--SQNGQFEKAVDDLNQDEESVTDMMDNLPDLITQCIQLDKKHSDRPELLQS-ETQL	232
Colobus_angolensis_palliatus	NAT--NQNGQFEKAVDDLNQDEESVTDMMDNLPDLITQCIQLDKKHSNRPELLQS-ETQL	232
golden_snub-nosed_monkey	NAT--NQNGQFEKAVDDLNQDEESVTDMMDNLPDLITQCIQLDKKHSDRPELLQS-ETQL	232
Mas_night_monkey	NET--NQNGRFEKALAGPNQDEESVTDIMDNLPDLITQCIQLDKKHSVRPELLQS-ETQF	89
common marmoset	NET--NQNGQFEKALAGPNQDEESVTDITDNLPDLITQCIQLDKKHSVRPELLQS-ETQF	155
Bolivian_squirrel_monkey	NET--NQNDQFEKALAGPNQDEDSVRGIMDSLPDLITQCIQLDKKHSVKPELLQS-DTQF	89
Philippine_tarsier	NET--NQGEQFQKGLADPIQDEESVTDNMDNLPDLITQCIQLDKKHSNRPELLQP-HTQL	234
gray_mouse_lemur	HET--NQSCHFKEGLDDSIQDEESVTDMMDNLPDLITECIQLDKKHSDRPELLQS-ETQL	232
Coquerels_sifaka	HET--SQSCQFQEGLADSIQDEESVTDMMDNLPDLITQCIQLDKKHSDRPELLQS-ETQL	232
small-eared_galago	HET--NQSGQFQEGLADPIQHEKDITDMMDNLPDLITQCIQLDKKHSNRPELLQS-GNQL	232
Damara_mole-rat	NKT--NQGEKFQERLSDPIQDEGNVTGMMRNPPDLIIQCIQLDKKYNDRPELLQS-ETQP	89
naked_mole-rat	NKT--NHREKFQERLSDPIQDEKSVTGMVGNLPDLIIQCIQLDKKYNDRPELLQS-EVRP	89
domestic_guinea_pig	NVT--NHKEKFHDRLCDHIQDEKSVTGMMENIPDLIIQCIQLDKKSNNKPELLQS-ETQL	89
long-tailed_chinchilla	NET--NHREKIHGRLADPIQDEKSATAMMENLPDLIIQCIQLDKKCNNKPELLQP-ENQL	121
degu	NET--NPKEKIQERLSDNIQDQKSVTAMMANLPDLIIQCIQLDKKCNEKPELLQS-EAQL	126
lesser_Egyptian_jerboa	NET--NQNDSLQEGLNDPIQDEE-ITDIMGNLPDLITQCIQLDKKLSNRPELLQS-ETHV	231
Ords_kangaroo_rat	NVTNTNQSNQFQEGLAGPIQVESSITDMMNNLPDLITQCIQLDKKY-DKPELLQL-ETQV	233
prairie_vole	SES--SQSTSFQKGQVEPTQDEE-ITDIMSNLPDLVTQCIQLDKKYKDRPELLQS-ESQV	230
Chinese_hamster	SET--SQSDQFQNRQDDPTHDEE-ITDIMGNLPDLITQCIQLDKKHKDRPELLQT-ETQV	230
golden_hamster	SET--SQSDQFQNRQADPTNDEE-ITDIMDNLPDLITQCIQLDKKHKDRPELLQT-ESQV	230
prairie_deer_mouse	SET--SQNDQFQKGQADPTHEEE-ITDIMGNLPDLITQCIQLDKNYKGRPELLQS-ESQV	230
house_mouse	RET--NQRDQFQNGQADPTQNEE-ITDIMDNLPDLITQCIQLDKKHKDRPELLQS-ESHV	230
Norway_rat	SET--DQRDQLQ---ADSTHDEE-ITDIMDNLPDLITQCIQLDKKHKDRPELLQS-ESHV	227
Upper_Galilee_mountains_blind_mole_rat	NDT--NQSDQFQEDISDDTHDEE-ITDIMGNLPDLIIQCIKLDQKRNDRPELLQS-ETQV	209
thirteen-lined_ground_squirrel	SET--NPSDQFQEGLADPIQDKESVADMMGNLPDLIIQCIQLDKKHNDRPELLQS-ETQL	221
rabbit	NEI--NKNDLFQEGLSDPMLDEESVTD-MDNLPDLITQCIQLEKKRNDRPELLQS-ETQL	231
American_pika	NEA--DQSDLFQEGLPVPMLVEESVTD-MDNLPDLITQCIQLEKERNHRPEILQS-QTQQ	231
Chinese_tree_shrew	NEA--NQSDQFQEGLADPIQNEESIMDMTDNLPDLITQCIQLEKKRSDRPELLQS-ETQL	232
Sunda_flying_lemur	NEI--SQNDQFQEGLANPIQDEESVTDMMDNLPDLITQCIQLDKKRSDRPELLQS-ETQL	232
dog	NEA--NQSDHLQEGLADSIHNEVSGTDMMNNLPDLITQCIQLDKKRNDRPELLQS-ETQF	224
domestic_ferret	NET--SQSDHFQEGIADSIQDDLSGTDMMDNLPDLITQCIKLDKKRSDRPELLQS-ETQL	223
Pacific_walrus	NET--NLSGNFQEGLADSIQDEVSGTDMMDNLPDLITQCIQLDKKQSDRPELLQS-ETQL	224
Weddell_seal	NET--NLSDNFQEGLADSIQDEVSGTDMMDNLPDLITQCIQLDKKHSDRPELLQS-ETQL	224
giant_panda	KET--DQSDHFQEGLPDSIQDEVSGRD-IDNLPDLITQCIQLDKKRSDRPELLQS-ETHL	223
polar_bear	KET--DQSDHFQEGLPDSIQDEVSGRD-IDNLPDLITQCIQLDKKRSDRPELLQS-ETHL	223
domestic_cat	NET--NQSDHFQEGLADPIQDEVSDTDMMDNLPDLITQCIQLDKKRSDRPELLQS-ETQL	217
Amur_tiger	NET--TQRDHFQEELADSIQDEVSDTDMMGNLPDLITQCIQLDKKRSDRPELLQS-ETQL	217
Balaenoptera_acutorostrata_scammoni	NET--NQSDHFQEELADSIQAEVSGTDLMDNLPDLITQCIQLDKQRSDRPELLQS-EAQI	89
killer_whale	NET--NQSDHFQEELADSIQAEVNGTDLMDNLPDLITQCIQLDKQRSDRPELLQP-EAQI	89
bottlenosed_dolphin	NET--NQSDHFQEELADSIQAEVNGTDLMDNLPDLITQCIQLDKQRSDRPELLQP-EAQI	89
Yangtze_River_dolphin	NES--NQSDHFQEELADSIQAEVSGTDLMDNLPDLITQCIQLDKQRSDRPELLQP-EAQI	89
sperm_whale	NET--NQSDHFQEELADSIQAEVSGTDLMDNLPDLITQCIQLDKQRSDRPELLQS-EAQI	89
chiru	DGF--NQSDHFQE-LTDSTQDEVSGPDPLDNLPDLITQCIQLDKKHSDWPELLES-EVQM	88
Bison_bison_bison	DGF--NQSDHFQEELADPTQDEVSGPDLLDNLPDLITQCIQLDKKHSEWPELLES-EVQM	89
cattle	DGF--NQSDHFQEELADPTQDEVSGPDLLDNLPDLITQCIQLDKKHSEWPELLES-EVQM	89
wild_yak	DGF--NQSDHFQEELADPTQDEVSGPDLLDNLPDLITQCIQLDKKHSEWPELLES-EVQM	89
water_buffalo	DGF--NQSDHFREDLADPTQDEVSGPDLLDNLPDLITQCIQLDKKHSDWPELLES-EVQM	89
goat	DGF--NQSDHFQEELTDSTQDEVSGPDPLDNLPDLITQCIQLDKKHSDWPELLES-EVQM	89
sheep	DGF--NQSDHFQEELTDSTQDEVSGPDPLDNLPDLITQCIQLDKKHSDWPELLES-EVQM	89
mouflon	DGF--NQSDHVQEELTDSTQDEVSGPDPLDNLPDLITQCIQLDKKHSDWPELLES-EVQM	89
pig	NET--NQSCHFQEGFADPIHAEVSGTDMMDNLPDLITQCIQVDKKRSNRPELLQS-EAQI	231
Bactrian_camel	NEP--DQSDHLQEGLGDSIQDEISGTDMMNNLPDLIIQCIQLDKKRSHRPELLPS-EAQT	232
Arabian_camel	NEP--DQSDHLQEGLGDSIQDEISGTDMMNNLPDLIIQCIHLDKKRSHRPELLPS-EAQT	232
Wild_Bactrian_camel	NEP--DQSDHLQEGLGDSIQDEISGTDMMNNLPDLIIQCIQLDKKRSHRPELLPS-EAQT	232
alpaca	NEP--DQSDHLQEGLGDSIQDEMSGTDMINNLPDLIIQCIQLDKKRSNRPELLPS-EAQT	232
black_flying_fox	NKA--YQTDLFQKVLADSFQDEVSGTDMMDNLPDLLSVFNRTRNVTTGQSCYSQRPSSQC	233
large_flying_fox	NKA--YQTDHFQKVLADSFQDEVSGTDMMDNLPDLLSVFNRTRNVTTGQSCYSQRPSSQC	233
big_brown_bat	NET--NESDHFQEGLPEPIQDEVSGTDMMDNLPDLITQCIQLDRKHSDKPELLQS-EATL	232
little_brown_bat	NET--SESDHFQEGLPDPIQDEVSGTDMMDNLPDLITQCIQLDRKHSDKPELLQS-EAQL	232
Brandts_bat	NET--SESDHFQEGLPDPIQDEVSGTDMMDNLPDLITQCIQLDRKHSDKPELLQS-EAQL	232
Myotis_davidii	NET--NESDHFQEGLPDPIQDEMSGTDMMDNLPDLITQCIQLDRKHSDKPELLQS-EAQF	232
European_shrew	NET--NQRDHSQEELPDSIEKEVSGTGIMDNLPDLITQCIQLDKKCNNKPELLQS-ETQL	225
star-nosed_mole	NET--NQCDYFQEGLVAPVEDEVSEKNMMDNLPDLITQCIQLDKKHSGRPELLRA-EEQL	231
horse	NET--NQTDHFQEGLADPIQDEVSGTDMVDNLPDLVTQCIQLDRKRSDRPELLQS-EPQL	226
Przewalskis_horse	NET--NQTDHFQEGLADPIQDEVSGTDMVDNLPDLVTQCIQLDRKRSDRPELLQS-EPQL	226
southern_white_rhinoceros	NET--NQSDHFQEGLADPIQDEVSGTDMMDNLPDLITQCIQLDKKRSDRPELLQS-EAQL	227
Cape_golden_mole	NEN--ILSDQLQEGLADPIQNEVSGRDIMENLPDLITQCIQLDRKCSDRPKLLQS-EAQL	230
Cape_elephant_shrew	NEI--IVSDQLQEGMAKPIHGEVSGADMMENLPDLIVQCIQLDRKHDDKPELLQP-QAQL	236
African_savanna_elephant	NKT--MLSDHLQEGLADPIQDEVSGTDMVENLPDLITQCIQLDRKRSDRPELLQS-EAHL	232
Florida_manatee	NKT--ILSDQLQEGLADPIQDEVSGTDMMENLPDLITQCIQLDRKRSDRPELLQS-EAQL	232
small_Madagascar_hedgehog	SDT--ILSDPLQEGLADPMQDEVSGIEIMENLPDLITQCMQLERKRSDRPELLQS-GAQI	226
Orycteropus_afer_afer	NEI--ILSDQLQEGLADPMQKEVSGTDMIENLPDLITQCIQLDRKHSNRPELLQS-EAQL	231

Fig. S1 (continued)

```
human                                   PLLASL-IQHQAL-FSPTDPPPKKGPIQLREGQLPLTPAKRARQQETQLCLYCSQSGHFT    290
chimpanzee                              PLLASL-IQHQAL-FSPTDPPPKKGPIHLQEGQLPLTPAKRARQQETQLCLYCSQSGHFT    290
pygmy_chimpanzee                        PLLASL-IQHQAL-FSPTDPPPKKGPIHLQEGQLPLTPAKRARQQETQLCLYCSQSGHFT    290
western_lowland_gorilla                 PLLASL-IQHQAL-FSPIDPPPKKGPIQLREGQLPLTPAKRARQQETQLCLYCSQSGHFT    262
Sumatran_orangutan                      PLLASL-IQHQAL-FSPTDPPPKKGPIQLREGQQPLTPAKRAHQQETQLCLYCSQSGHFT    286
northern_white-cheeked_gibbon_a         ------------------------------------------------------------    186
northern_white-cheeked_gibbon_b         PLLASL-IQHQAF-FSPTDPPPKKGPIQLREGQLPLTPAKRAHQLETHLCLYCSQCGHFT    90
sooty_mangabey                          PLLASL-IQHQAL-FSPTDPPPKKGPIQLREGQLPLTPAKRARQQETQLCLYCSQSGHFT    290
green_monkey                            PLLASL-IQHQAL-FSPTDPPPKKGPIQLREGQLPLTPGKRARQQETQLCLYCSQSGHFT    290
crab-eating_macaque                     PLLASL-IQHQAL-FSPTDPPPKKGPIQLREGQLPLTPAKRARQQETQLCLYCSQSGHFT    290
rhesus_monkey                           PLLASL-IQHQAL-FSPTDPPPKKGPIQLREGQLPLTPAKRARQQETQLCLYCSQSGHFT    290
pig-tailed_macaque                      PLLASL-IQHQAL-FSPTDPPPKKGPIQLREGQLPLTPAKRARQQETQLCLYCSQSGHFT    290
drill                                   PLLASL-IQHQAL-FSPTDPPPKKGPIQLREGQLPLTPAKRARQQETQLCLYCSQSGHFT    290
olive_baboon                            PLLASL-IQHQAL-FSPTDPPPKKGPIQLREGQLPLTPAKRARQQETQLCLYCSQSGHFT    290
Colobus_angolensis_palliatus            PLLASL-IQHQAL-FSPTDPPPKKGPIQLREGQLPLTPAKRARQQETQLCLYCSQPGHFT    290
golden_snub-nosed_monkey                PLLASL-IQHQAL-FSPTDPPPKKGPIQLREGQLPLTPAKRARQQETQLCLYCSQSGHFT    290
Mas_night_monkey                        PLLTSL-IQHQAL-VSPTDPPPKKGPMQLREGQLPLTPAKRAYQQETQLCLYCNQSGHFT    147
common marmoset                         PLLPSL-IQHQAL-VSPTDPPPKRGPMQLREGQLPLTPAKRAYQQETQLCLYCNQSGHFT    213
Bolivian_squirrel_monkey                PMLTSL-IQHQAL-VSPTDPPPKKGPMQLREGQLPLTPAKRAYQQETQLCLYCNQSGHFT    147
Philippine_tarsier                      PMLASP-IHHRAL-SSPSGPPHKEEPILLRGGQMPLTPAKRARQQENQLCLYCSQAGHFT    292
gray_mouse_lemur                        PMLASL-IQNPAL-SSSTGPPPKEEPIQLRGSQPPLTPAKRARQQETQLCLYCSQADHFT    290
Coquerels_sifaka                        PMLASL-IQNPPL-SSFTGPPSKEEPVQLRGGQPPLTPAKRARQQETQLCLYCSQAGHFT    290
small-eared_galago                      SVRDPL-IHHQAL-SSSIGLPPKEEPIQQR-SQPPLTPAKRARQQEIQLCLYCSQSGHFT    289
Damara_mole-rat                         PLLASL-IHHQSL-SSSIGSPSKQEPIQLRGGQLPLTPAKRAHQQETQLCLYCSQAGHFT    147
naked_mole-rat                          PLLGSL-IPHQYL-SSSTGPPPKEEPIKLRGGQQPLTPAKRAHQQETQLCLYCSQAGHFT    147
domestic_guinea_pig                     PVLASL--------SSSTSPRPKEETIQLRGGQLPLTPAKRARQQETQLCLYCSQAGHFT    141
long-tailed_chinchilla                  PVLEFL-NHHQSS-SRSTGPPPKEEPIQLRGGQLPLTPAKRARQQESPLCLYCSQAGHFT    179
degu                                    PELAFP-NHPPSF-SSSMCPPPKEEPIQLRGG-LPLTPAKRARQQESQLCLYCSQAGHFT    183
lesser_Egyptian_jerboa                  PTFASL-IHHQSIIPVPAAPPSKEESTQVRGAQLPLTPAKRVRQQETQLCLYCSQADHFT    290
Ords_kangaroo_rat                       PELPSL-IYDKSL-SRPTVLPSKEEHTQLRGGHRLLTPAKRARQQETQLCLYCSQAGHFT    291
prairie_vole                            PMFSSTSNHHLSF-LSPIGLQAKDEPKQIQGAQLPVTPAKRARQQETQLCLYCSQAGHFT    289
Chinese_hamster                         PMFSST-NHHQSF-LGLMGPLPKDELKPFQGAQMPVTPAKRARQQETQLCLYCSQAGHFT    288
golden_hamster                          PVFSST-NHRQSF-LSPMVPLPKDEFKSLQGAQMPVTPAKRARQQETQLCLYCSQAGHFT    288
prairie_deer_mouse                      PIFAST-TKHQSF-FSPKGPLPKDEPKQFQGAQPPVTPAKRARQQETQLCLYCSQAGHFT    288
house_mouse                             PMFAST-NHYQSF-IGPVRPLPKDGPRQLQGAHLPVTPAKRARQQETQLCVYCNQAGHFT    288
Norway_rat                              PMVASP-NCSQSF-LSPIRPLPKDGLKPMQGAHLPVTPAKRARQQETQLCLYCNQAGHFT    285
Upper_Galilee_mountains_blind_mole_rat  SMFSSM-LHQQSF-LSPIRLAPKEEPTQLRGMQLPVTPAKRARQQETQSCLYCSQAGHFT    267
thirteen-lined_ground_squirrel          PTLASL-TYHQSL-SSTTGLPSREEPSQLRGGQLPLTPAKRARQQETHLCLYCNQADHFT    279
rabbit                                  PVLASL-IHHQAL-SSSIGLPPKEEPTQLRGGQLPLTPAKRARQQETQLCLYCSQSGHFA    289
American_pika                           PVLASS-IHHEAL-SCSLIPSTKEEPIQLRVGQLPLTPAKRARQQDTQLCLYCSQSGHFT    289
Chinese_tree_shrew                      PMLAPL-IHYQAL-SSCTGLPPKEKPIQLRGCQPPLTPHLVNP*----------------    273
Sunda_flying_lemur                      PMLASL-IHNQAL-SSPTCLLPKEEPIQLRGGQPPLTPAKRARQQETKLCLYCNQAGHFT    290
dog                                     PMLASL-IHHQAF-SSPTNLPPKEEPIQLRGSQPPLTSAKRARQQEAQLCLYCSQAGHFT    282
domestic_ferret                         PVLDCM-IHHQAF-SIPTDLPPKEEPIQLRGSQPPLTPAKRARQQEAQLCLYCSQAGHFT    281
Pacific_walrus                          PMLASM-IHPQAF-SSPTDLPPKEEPIQLRGSQPPLTPAKRALQQEAQLCLYCSQAGHFT    282
Weddell_seal                            PMLASM-IHHQAF-SSPTDLPPKEEPIQLRGSQPPLTPAKRALQQEAQLCLYCSQAGHFT    282
giant_panda                             PMFASM-IHHQAF-SGPTDLPPKEEPIQLRGSQPPLTPAKRGRQQEAQLCLYCSQAGHFT    281
polar_bear                              PMLASM-IHHQAF-SSPTDLPPKEEPIQLRGSQPPLTPAKRARQQEAQLCLYCSQAGHFT    281
domestic_cat                            PVLASL-IQHQPL-SSPTDVSAKEEPIQLRGSQPPLTPAKRARQQEAQLCLYCSQAGHFT    275
Amur_tiger                              PVLASL-IQHQPF-SSPTDVSAKEEPIQLRGSQPPLTPAK*-------------------    255
Balaenoptera_acutorostrata_scammoni     PVLASL-IRHQAL-SNPTGPPPKEDPIQLRGSQPPLTPAKRARQQETQSCLYCSQAGHFT    147
killer_whale                            PVLASL-I------SNPTGPPPKEDPIQLRGSQLPLTPAKRARQQETQSCLYCSQAGHFT    142
bottlenosed_dolphin                     PVLASL-I------SNPTGPPPKEDPIQLRGSQLPLTPAKRARQQETQSCLYCSQAGHFT    142
Yangtze_River_dolphin                   PVLASL-IHHQAL-SNPTGPPPKEDPIQLRGSQLPLTPAKRARQQETQSCLYCSQAGHFT    147
sperm_whale                             PVLASL-IHHQAL-SNPTGPPPKEDPIQLRGSQPPLTPAKRARQQETQSCLYCSQAGHFT    147
chiru                                   PVLAHW-IHHQAF-SNPTGTAPKEDPIQLRGSQPPLTPAKRAHQQEVQACLYCSQAGHLT    146
Bison_bison_bison                       PVLARW-IHHQAF-SNPTGPAPKEDRVQLRGSQPPLTPAKRARQQEVQACLYCSQAGHVT    147
cattle                                  PVLARW-IHHQAF-SNPTGPAPKEDRIQLRGSQPPLTPAKRARQQEVQACLYCSQAGHVT    147
wild_yak                                PVLARW-IHHQAF-SNPTGPAPKEDRIQLRGSQPPLTPAKRARQQEVQACLYCSQAGHVT    147
water_buffalo                           PVLARW-IHHQAF-SNPTGPAPKEDRIQLRGSQPPLTPAKRARQQEVQACLYCSQAGHLT    147
goat                                    PVLARW-IHHQAF-SNPTGPAPKEDPIQLRGSQPPLTPAKRAHQQEVQACLYCSQAGHLT    147
sheep                                   PVLARW-IHHQAF-SNPTGPAPKEDPIQLRGNQPPLTPAKRAHQQEVQACLYCSQPGHLT    147
mouflon                                 PVLARW-IHHQAF-SNPTGPAPKEDPIQLRGNQPPLTPAKRAHQQEVQACLYCSQPGHLT    147
pig                                     PVLASL-IHHQTL-SSTTGPPPKEDPVQLRGGQPPLTPAKRARQQETHSCLYCSQSGHFT    289
Bactrian_camel                          SMLASL-VHHQAL-FSPRGPPSKEDPIHLRRSQLPLTPAKRARQQETQLCLYCGQAGHFT    290
Arabian_camel                           SMLASL-VHHQAL-FSPRGPPSKEDPIQLRRSQLPLTPAKRARQQETQLCLYCGQAGHFT    290
Wild_Bactrian_camel                     SMLASL-VHHQAL-FSPRGPPSKEDPIHLRRSQLPLTPAKRARQQETQLCLYCGQAGHFT    290
alpaca                                  SMLASL-VHHQAL-FSPRGPPSKEDPIQLRRSQLPLTPAKRARQQETQLCLYCGQAGHFT    290
black_flying_fox                        CLLIPT-IKSPS----QVHHPWKRLYSCKEASHLSLRPNEP--TRRNTVCLYCS*-----    280
large_flying_fox                        CLLIPT-IKSPS----QVHHPWKRLYSCKEASHLSLRPNEP--TRRNTVCLYCS*-----    280
big_brown_bat                           PVLSSL-IHYQTL-SSPSCPSSK-ELIQLRGSQPSLTPAKRARQQETQLCLYCSQADHFT    289
little_brown_bat                        PVLSSL-VHYQTL-SSPSCPSSK-ELIQLRGSQPPLTPAKRARQQETHLCLYCSQADHFT    289
Brandts_bat                             PVLSSL-VHYQTL-SSPSCPSSK-ELIQLRGSQPPLTPAKRARQQETHLCLYCSQADHFT    289
Myotis_davidii                          PVLSSL-VHYQTL-SSPSCPSSK-ELIQLRGSQPPLTPAKRARQQETHLCLYCSQADHFT    289
European_shrew                          PMLTSL-VCNQAL-YNPK-DSCKENSIQLHGGHPLRTPVKRAHQQETHLCLYCSQSDHFT    282
star-nosed_mole                         TVLASH-SHHQAL-SSPTDPLPKDEMIQLRGSQSPLTPAKRARQQEAHLCLYCSQAGHIT    289
horse                                   PMLASL-INHQAL-SSPIGLSPKEEPIQLRGSQLPLTPAKRARQQETQLCLYCSQSGHFT    284
Przewalskis_horse                       PMLASL-INHQAL-SSPIGLSPKEEPIQLRGSQLPLTPAKRARQQETQLCLYCSQSGHFT    284
southern_white_rhinoceros               PKLASL-IHHQAL-SSPTGLSPKEESIQLRGSQLPLTPAKRARQQETRLCLYCSQSGHFT    285
Cape_golden_mole                        PRLASP-IHHQHY-SIPIGPPIKETSIQLRGGQLPLTPAKRVHQQDNQLCLYCSQPGHFT    288
Cape_elephant_shrew                     P------IHHQYV-PSLTGPPAKEEPIQLRGGQLPLTPAKRARQQETELCLYCSQPGHFT    289
African_savanna_elephant                LRPASL-IHHQYL-SNPTGPPAKEEPTQLRGGQQPLTPAKRARQQETQFCLYCSQPGHFT    290
Florida_manatee                         PRLASL-VHHQYL-SSPTGPPAKEEPTQLRGGQLPLTPAKRARQQETQFCLYCSQPGHFT    290
small_Madagascar_hedgehog               PSLDSS-L-HPYI-STLTGPPAKKEPIQLRGGQFPLTPAKRARQQETKWCLYCSQPNHFT    283
Orycteropus_afer_afer                   PRLPSL-IHHQYQ-SRPTGPPTKEEPIQLRGGQLPLTPAKRARQQETQLCLYCSQPGHVT    289
```

Fig. S1 (continued)

human	RDCLAKRSRAPATTNNTAHQ*--------	310
chimpanzee	RDCLAKRSQAPATTNNTAHQ*--------	310
pygmy_chimpanzee	RDCLAKRSRAPATTNNTAHQ*--------	310
western_lowland_gorilla	RDCLAKRSRAPAMTNNTAHQ*--------	282
Sumatran_orangutan	RDCLARRSRAPATTNNTAHQ*--------	306
northern_white-cheeked_gibbon_a	-----------------------------	186
northern_white-cheeked_gibbon_b	RDCLAKRS*--------------------	98
sooty_mangabey	RDCLAKRSRAPATTNNTAHQ*--------	310
green_monkey	RDCLAKRSRTPAMTNNTAHQ*--------	310
crab-eating_macaque	RDCLAKRSRAPAMTNNTAHQ*--------	310
rhesus_monkey	RDCLAKRSRAPAMTNNTAHQ*--------	310
pig-tailed_macaque	RDCLAKRSRAPAMTNNTAHQ*--------	310
drill	EDCLAKRSRAPATTNNTAHQ*--------	310
olive_baboon	RDCLAKRSRAPATTNNTAHQ*--------	310
Colobus_angolensis_palliatus	RDCLAKRSRAPAMTNNTAHQ*--------	310
golden_snub-nosed_monkey	RDCLAKRSRAPATTNNTAHQ*--------	310
Mas_night_monkey	RDCLAKRSRALAMTNNTAHQ*--------	167
common marmoset	RDCLAKRSRALAMTNNSSTVRRDQESHF*	241
Bolivian_squirrel_monkey	RDCLAKRSRALAVTNNTAHQ*--------	167
Philippine_tarsier	RDCLAKRSRAPARTNDIAHQ*--------	312
gray_mouse_lemur	RDCFAKRSRAPARTNNTTHQ*--------	310
Coquerels_sifaka	RDCLAKRSRAPARTNNTTHQ*--------	310
small-eared_galago	RDCLAKRSRTPTRTNNTTHQ*--------	309
Damara_mole-rat	RDCLAKRSRAPERTNDTAHQ*--------	167
naked_mole-rat	RDCLAKRSRAPERTDNTAHQ*--------	167
domestic_guinea_pig	RDCLAKRSRAPARTNNPAYQ*--------	161
long-tailed_chinchilla	RDCLAKRSRAPAWTNNPAHQ*--------	199
degu	KDCLAKRSRAPARTNNPDHQ*--------	203
lesser_Egyptian_jerboa	RDCLAKRSRTPSKTKYTTHQ*--------	310
Ords_kangaroo_rat	EDCLAKRSRSPSTDNLA-HQ*--------	310
prairie_vole	RECLAKRSRTPARRKM*------------	305
Chinese_hamster	RECLAKRSRTPARRKNVVH*---------	307
golden_hamster	RECLAKRSRTPARRKM*------------	304
prairie_deer_mouse	RECLAKRSRTPARRKM*------------	304
house_mouse	RDCLAKRSRTPARKKM*------------	304
Norway_rat	RDCLAKRSRTPARKKM*------------	301
Upper_Galilee_mountains_blind_mole_rat	RDCLAKRSRTPASVKNATNK*--------	287
thirteen-lined_ground_squirrel	RDCLAKRSRAPASTNNPDHQ*--------	299
rabbit	RDCLAKRSRAPARTNNSPYQ*--------	309
American_pika	RDCLAKRSRAPARTNNPPFQ*--------	309
Chinese_tree_shrew	-----------------------------	273
Sunda_flying_lemur	RDCLAKRSRAPVRANNPAHQ*--------	310
dog	RDCLAKRSRAPARINNSTHQ*--------	302
domestic_ferret	RDCLAKRSRAPARINNPTHQ*--------	301
Pacific_walrus	RDCLAKRSRAPARINNPTHQ*--------	302
Weddell_seal	SDCLAKRSRAPARINNPTHQ*--------	302
giant_panda	RDCLAKRSRAPARINNPTHQ*--------	301
polar_bear	RDCLAKRSRAPARINNPTHQ*--------	301
domestic_cat	RDCLAKRSRAPARINNPTRQ*--------	295
Amur_tiger	-----------------------------	255
Balaenoptera_acutorostrata_scammoni	RDCLAKRSRAPARINNPAHQ*--------	167
killer_whale	RDCLAKRSRAPARINNPAHQ*--------	162
bottlenosed_dolphin	RDCLAKRSRAPARINNPAHQ*--------	162
Yangtze_River_dolphin	RDCLAKRSRAPARINNPAHQ*--------	167
sperm_whale	RDCLAKRSRAPARINNPAHQ*--------	167
chiru	LDCLAKRSRAPARINNPTHQ*--------	166
Bison_bison_bison	LDCLAKRSRAPERINNPTHQ*--------	167
cattle	LDCLAKRSRAPERINNPTHQ*--------	167
wild_yak	LDCLAKRSRAPERINNPTHQ*--------	167
water_buffalo	LDCLAKRSRAPARINNPTHQ*--------	167
goat	LDCLAKRSRAPARINNPTHQ*--------	167
sheep	LDCLAKRSRAPARINNPTHQ*--------	167
mouflon	LDCLAKRSRAPARINNPTHQ*--------	167
pig	KDCLAKRSRAPTRTNNSSQQ*--------	309
Bactrian_camel	RDCLAKRFRAPARTNNPAYQ*--------	310
Arabian_camel	RDCLAKRFRAPARTNNPAYQ*--------	310
Wild_Bactrian_camel	RDCLAKRFRAPARTNNPAYQ*--------	310
alpaca	RDCLAKRFRAPARTNNPAYQ*--------	310
black_flying_fox	-----------------------------	280
large_flying_fox	-----------------------------	280
big_brown_bat	RDCLAKRSRAPARINNPAHQ*--------	309
little_brown_bat	RDCLAKRSRAPARINNPAHQ*--------	309
Brandts_bat	RDCLAKRSRAPARINNPAHQ*--------	309
Myotis_davidii	RDCLAKRSRAPARINNPAHQ*--------	309
European_shrew	RDCLAKRFRAPQRINNPTHK*--------	302
star-nosed_mole	SDCLAKRSRAPARISNPTHQ*--------	309
horse	RDCLAKRSRAPARINDPAHQ*--------	304
Przewalskis_horse	RDCLAKRSRAPARINDPAHQ*--------	304
southern_white_rhinoceros	RDCLAKRSRAPARINNPAHQ*--------	305
Cape_golden_mole	KACLAKRSRAPARTNNPTHQ*--------	308
Cape_elephant_shrew	ADCLAKRSRALTRTNNSTHQ*--------	309
African_savanna_elephant	KDCLAKRSRALARKNNPTHK*--------	310
Florida_manatee	KDCLAKRSRAPARSNNPTHQ*--------	310
small_Madagascar_hedgehog	KDCLAKRSRAPARTSKPTHQ*--------	303
Orycteropus_afer_afer	KDCLAKRSRAPARTNDPTHQ*--------	309

Fig. S1 (continued)

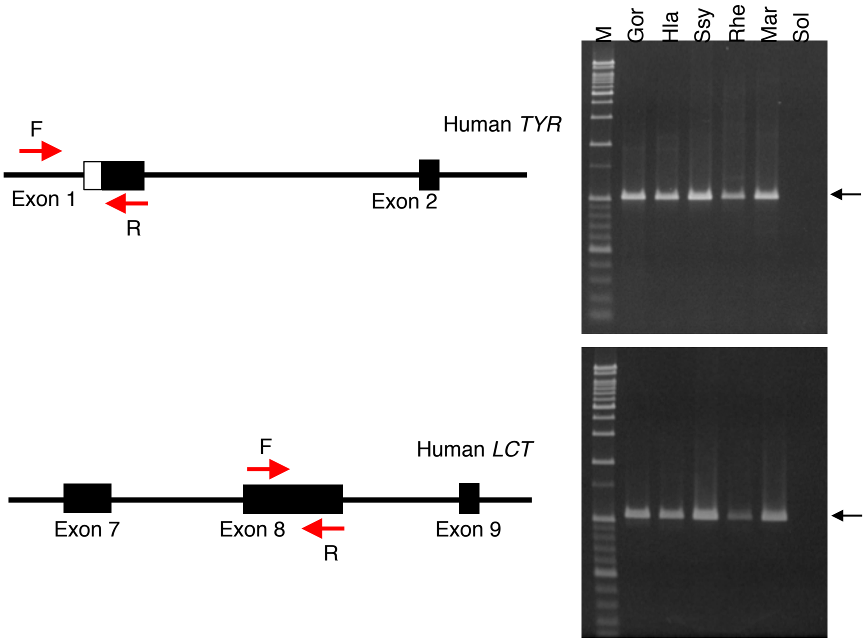

Human TYR
F
R
Exon 1
Exon 2
Human LCT
F
R
Exon 7
Exon 8
Exon 9
M
Gor
Hla
Ssy
Rhe
Mar
Sol

Fig. S2

```
pig                                   GTAGTGGACAGCAGTTTTTTGCATTTCTTTCACTTGGTTCTCAGCATACCCTTGTGACTTCTTTATAGCCAACACCTTTGGCTACTTGCTCATGACACTGCATCTCTGATCATAGAGCTA
killer_whale                          GTAGTGGACAACAGTTTTTGGCATTT------CTCAGCTCTCAGCATACCCTTGTGACTTCTTCATAGTTAACAGATTTGGCTACTTGCTCATGACACCGTCTGTCTGATCACAGAGCAA
bottlenosed_dolphin                   GTAGTGGACAACAGTTTTTGGCATTT------CTCAGCTCTCAGCATACCCTTGTGACTTCTTCATAGTTAACAGATTTGGCTACTTGCTCATGACACCGTCTGTCTGATCACAGAGCAA
Yangtze_river_dolphin                 GTAGTGGACAACAGTTTTTGGCATTT------CTCAGCTCTCAACATACCCTTGTGACTTCTTCATAGTTAACAGATTTGGCTACTTGCTCATGACACCGTCTGTCTGATCACAGAGCAA
sperm_whale                           GTAGTGGACAACAGTTTTTGGCATTT------CTCAGCTCTCAGCATACCCTTGTGACTTCTTCATAGCTAACAGATTTGGCTACTTGCTCATGACACCGTCTCTCTGATCACAGAGCAA
Balaenoptera_acutorostrata_scammoni   GTAGTGGACAACAGTTTTTGGCATTT------CTCAGCTCTCAGCATACCCTTGTGACTTCTTCATAGCTAACAGATTTGGCTACTTGCTCATGACACCGTCTCTCTGATCACAGAGCAA
water_buffalo                         ATGGTGGACAACAATTTTGAGCATTT------CTCAGCTGTCAGCATACATTTGTGATCTC---ATAGCTAACAGCTTTGTCTACTTTCTCATGATGCCATTGCTCTGATCACAGAGTAA
Bison_bison_bison                     ATGGTGGACAACAATTTTGAGCATTT------CTCAGCTGTCAGCATACATTTGTGACCTC---ATAGCTAACAGCTTTGTCTACTTTCTCATGATGCCATTGCTCTGATCACAAAGTAA
cattle                                ATGGTGGACAACAATTTTGAGCATTT------CTCAGCTGTCAGCATACATTTGTGATCTC---ATAGCTAACAGCTTTGTCTACTTTCTCATGATGCCATTGCTCTGATCACAAAGTAA
wild_yak                              ATGGTGGACAACAATTTTGAGCATTT------CTCAGCTGTCAGCATACATTTGTGATCTC---ATAGCTAACAGCTTTGTCTACTTTCTCATGATGCCATTGCTCTGATCACAAAGTAA
chiru                                 ATGGTGGACAACAATTTTGAGCATTT------CTCAGCTGTCAGCATACATTTGTGACCTC---ATGGCTAACAGCTTTGTCTACTTTCTCATGACGCCGTTGCTCTGATCACAGAGTAA
goat                                  ATGGTGGACAACAATTTTGAGCATTT------CTCATCTGTCAGCATACATTTGTGACCTC---ATGGCTAACAGCTTTGCCTACTTTCTCATGATGCTGTTGCTCTGATCACAGAGTAA
sheep                                 ATGGTGGACAACAATTTTGAGCATTT------CTCATCTGTCAGCATACATTTGTGACCTC---GCGGCTAACAGCTATGCCTACTTTCTCATGATGCTGTTGCTCTGATCACAGAGTAA
mouflon                               ATGGTGGACAACAATTTTGAGCATTT------CTCATCTGTCAGCATACATTTGTGACCTC---GCGGCTAACAGCTATGCCTACTTTCTCATGATGCTGTTGCTCTGATCACAGAGTAA

pig                                   AACTACTGTCTGCCTGTTGGTCTCTGGTACTGGTACTCAATTCTGCTTAGTCCAACTTGATTCCAGAAGATCCAATGGAGAATTATATAGACTCACCACCTACCTTACAGGTAGAGCATT
killer_whale                          AACCACTGTCTGCTCATTGGTATCCAATATTGGTACTCAATTCTGCTTAGTCTCACTTGATTCCAGAAGATACAGTAGAGAATTACACAGAATCACCACCT-------------------
bottlenosed_dolphin                   AACCACTGTCTGCTCATTGGTATCCAATATTGGTACTCAATTCTGCTTAGTCTCACTTGATTCCAGAAGATACAGTAGAGAATTACACAGAATCACCACCT-------------------
Yangtze_river_dolphin                 AACCACTGTCTGCTCATTGGTATCCAATATTGGTACTCAATTCTGCTTAGTCTCACTTGATTCCAGAAGATACAGTAGAGAATTACACAGAATCACCACCT-------------------
sperm_whale                           AACCACTGTCTGCCCATTGGTATCCAATATTGGTACTCAATTCTGCTTAGTCTCACTTGATTCCAGAAGATACAGTAGAGAATTACACAGAATCACCACCT-------------------
Balaenoptera_acutorostrata_scammoni   AACCACTGTCTGCCCATTGGTATCCAATATTGGTACTCAATTCTGCTTAGTCTCACTTGATTCCGGAAGATACAGTAGAGAATTACACAGAATCACCACCT-------------------
water_buffalo                         ATTCACTGTCTGCCCACTAGTATCCAGTACTGATACTCAGTTCTGCCTAGGCCCACTTGATTGCAGAAGATACAATAGACAATTACAGGGAATCACCAACTATC----------------
Bison_bison_bison                     ATCCACTGTCTGCCCACTAGTATCCAGTACTGATACTCAATTCTGCCTAGGCCCACTTGATGGCAGAAGATACAATAGACAATTACAGGGAATCACCAACTATC----------------
cattle                                ACCCACTGTCTGCCCACTAGTATCCAGTACTGATACTCAATTCTGCCTCGGCCCACTTGATGGCAGAAGATACAATAGACAATTACAGGGAATCACCAACTATC----------------
wild_yak                              ATCCACTGTCTGCCCACTAGTATCCAGTACTGATACTCAATTCTGCCTAGGCCCACTTGATGGCAGAAGATACAATAGACAATTACAGGGAATCACCAACTATC----------------
chiru                                 ATCCACTGTCTACCCACTAGTATCCAGTACTGATACTCAATTCTGCCTAGTCCCACTTGCTTGCAGAAGATACAATAGACAATTACAGGGAATCACCAACTATC----------------
goat                                  ATCCACTGTCTACCCACTAGTATCCAGTACTGGTACTCAATTCTGCCTAGTCCCACTTGCTTGCAGAAGATACAATAGACAATTACAGGGAATCACCAACTATC----------------
sheep                                 ATCCACTGTCTACCCACTAGTATCCAGTACTGGTACTCAATTCTGCCTAGTCCCACTTGCTTGCAGAAGATACAATAGACAATTACAGGGAATCACCAACTATC----------------
mouflon                               ATCCACTGTCTACCCACTAGTATCCAGTACTGGTACTCAATTCTGCCTAGTCCCACTTGCTTGCAGAAGATACAATAGACAATTACAGGGAATCACCAACTATC----------------

pig                                   CTTCCCTTCAGGCAGAGAATCTGATTCTGCAGCCACAAATGCAGCATGTGACTGAGGAGAACCCTGCTCTAAGGGGCCAAGTCATGTCTGCCCTAACCCCACCAATGATGCCTGTACCCT
killer_whale                          ------------------------------------------------------------------------------------------------------------------------
bottlenosed_dolphin                   ------------------------------------------------------------------------------------------------------------------------
Yangtze_river_dolphin                 ------------------------------------------------------------------------------------------------------------------------
sperm_whale                           ------------------------------------------------------------------------------------------------------------------------
Balaenoptera_acutorostrata_scammoni   ------------------------------------------------------------------------------------------------------------------------
water_buffalo                         ------------------------------------------------------------------------------------------------------------------------
Bison_bison_bison                     ------------------------------------------------------------------------------------------------------------------------
cattle                                ------------------------------------------------------------------------------------------------------------------------
wild_yak                              ------------------------------------------------------------------------------------------------------------------------
chiru                                 ------------------------------------------------------------------------------------------------------------------------
goat                                  ------------------------------------------------------------------------------------------------------------------------
sheep                                 ------------------------------------------------------------------------------------------------------------------------
mouflon                               ------------------------------------------------------------------------------------------------------------------------

pig                                   GCTCACTTGAGCATCTTTCCCAATTTCATGGTGACCCCACCAATATCTCAGAGTTTCTTGCTCAGATGACTACCTACCTGACAACTTTGAAGATCCCCAATCCTGAAGATAATGCTCATG
killer_whale                          ------------------------------------------------------------------------------------------------------------------------
bottlenosed_dolphin                   ------------------------------------------------------------------------------------------------------------------------
Yangtze_river_dolphin                 ------------------------------------------------------------------------------------------------------------------------
sperm_whale                           ------------------------------------------------------------------------------------------------------------------------
Balaenoptera_acutorostrata_scammoni   ------------------------------------------------------------------------------------------------------------------------
water_buffalo                         ------------------------------------------------------------------------------------------------------------------------
Bison_bison_bison                     ------------------------------------------------------------------------------------------------------------------------
cattle                                ------------------------------------------------------------------------------------------------------------------------
wild_yak                              ------------------------------------------------------------------------------------------------------------------------
chiru                                 ------------------------------------------------------------------------------------------------------------------------
goat                                  ------------------------------------------------------------------------------------------------------------------------
sheep                                 ------------------------------------------------------------------------------------------------------------------------
mouflon                               ------------------------------------------------------------------------------------------------------------------------
```

Fig.S3

```
pig                                 TCAAGTTCATTTTTGACCACCTATCTCAGCAGATGGAAAGCTGTGGGGTCATATCTGGGCCTGACCAGAGTGCTCTGTTGAAGCAACATGAGAACTTTGTTCTTGAGTTCCAGCAGTCAT
killer_whale                        ---------------------------------------------------------------ACCAGAGTGCTCTGTTGAAGCAATATGAGAACTTTGTTCTTGAGTTACAGCAGTCAT
bottlenosed_dolphin                 ---------------------------------------------------------------ACCAGAGTGCTCTGTTGAAGCAATATGAGAACTTTGTTCTTGAGTTACAGCAGTCAT
Yangtze_river_dolphin               ---------------------------------------------------------------ACCAGAGTGCTCTGTTGAAGCAATATGAGAACTTTGTTCTTGAGTTACAGCAGTCAT
sperm_whale                         ---------------------------------------------------------------ACCAGAGTGCTCTGTTGAAGCAATATGAGAACTTTGTTCTTGAGTTACAGCAGTCAT
Balaenoptera_acutorostrata_scammoni ---------------------------------------------------------------ACCAGAGTGCTCTGTTGAAGCAATATGAGAACTTTGTTCTTGAGTTACAGCAGTCAT
water_buffalo                       ---------------------------------------------------------------ACCA-------TTGTTGAAGCAGAATGAGAACTTTCTTCCCAAGTTCCAGCAGTCAT
Bison_bison_bison                   ---------------------------------------------------------------ACCA-------TTGTTGAAGCAGAATGAGAACTTTCTTCCCAAGTTCCAGCAGTCAT
cattle                              ---------------------------------------------------------------ACCA-------TTGTTGAAGCAGAATGAGAACTTTCTTCCCAAGTTCCAGCAGTCAT
wild_yak                            ---------------------------------------------------------------ACCA-------TTGTTGAAGCAGAATGAGAACTTTCTTCCCAAGTTCCAGCAGTCAT
chiru                               ---------------------------------------------------------------ACCG-------TTGTTTAAGCAGAATGAGAACTTTCTTACCAAGTTCCAGCAGTCAT
goat                                ---------------------------------------------------------------ACCA-------TTGTTGAAGCAGAATGAGAACTTTCTTACCAAGTTCCAGCAGTCAT
sheep                               ---------------------------------------------------------------ACCA-------TTGTTGAAGCAGAATGAGAATTTTCTTACCAAGTTCCAGCAGTCAT
mouflon                             ---------------------------------------------------------------ACCA-------TTGTTGAAGCAGAATGAGAATTTTCTTACCAAGTTCCAGCAGTCAT


pig                                 TTGGTGAGCTCACAAAACAGGAAATGAAGTCCCTGGTGA---CTAAGGTTGACAAAGGGGACAAATTCTCCCAGCAGGATACTACAACTTTCCAGCTCCTTGCTCAGAATCTGAGATATA
killer_whale                        TTGGTGAGCCCACAAAACAGGAAATGAACCCCTTGATGAATGCTAAGGTTGACAAAAGGGACAACTTCTCTCAGCAGGATGCCACTACTTTCCAGCTCCTTGCTCAAAATCTGAGATATA
bottlenosed_dolphin                 TTGGTGAGCCCACAAAACAGGAAATGAACCCCTTGATGAATGCTAAGGTTGACAAAAGGGACAACTTCTCTCAGCAGGATGCCACTACTTTCCAGCTCCTTGCTCAAAATCTGAGATATA
Yangtze_river_dolphin               TTGGTGAGCCCACAAGACAGGAAATGAACCCCTTGGTGAATGCTAAGGTTGACAAAAGGGACGACTTCTCTCAGCAGGATGCCACTACTTTCCAGCTCCTTGCTCAAAATCTGAGATATA
sperm_whale                         TTGGTGAGCCCACAAAACAGGAAATGAACCCCTTGGTGAATGCTAAGGTTGACAAAAGGGACAACTTCTCTCAGCAGGATGCCACTACTTTCCAACTCCTTGCTCAAAATCAGAGATATA
Balaenoptera_acutorostrata_scammoni TTGGTGAGCCCACAAAACAGGAAATGAACCCCTTGGTGAATGCTAAGGTTGACAAAAGGGACAACTTCTCTCAGCAGGATGCCACTACTTTCCATCTCCTTGCTCAAAATCTGAGATATA
water_buffalo                       TTGATGAGCCCACAAAACAGGAAATGAACCACCTGGTGAATACTAAGATTGACAAAGAGGACAACTTCTCTCAGCAAAATGCCAGTACGTTCCACCTCCTTGCTCAGAATCTGAGATATG
Bison_bison_bison                   TTGATGAGCCCACAAAACAGGAAATGAACCGCCTGGTGAATACTAAGGTTGACAAAGAGGACAACTTCTCTCAGCAAAATGCCAGTACATTCCACCTCCTTGCTCAGAATCTGAGATATG
cattle                              TTGATGAGCCCACAAAACAGGAAATGAACCGCCTGGTGAATACTAAGGTTGACAAAGAGGACAACTTCTCTCAGCAAAATGCCAGTACATTCCACCTCCTTGCTCAGAATCTGAGATATG
wild_yak                            TTGATGAGCCCACAAAACAGGAAATGAACCGCCTGGTGAATACTAAGGTTGACAAAGAGGACAACTTCTCTCAGCAAAATGCCAGTACATTCCACCTCCTTCCTCAGAATCTGAGATATG
chiru                               TTGATGAGCCCACAAAACAGAAAATGAACCGCCTGGTGAATACTAAGGTTGACAAAGAGGACAACTTCTCTCAGCAAAATGCCAGTATGTTCCACCTCCTTGCTCAGAATCTGAGATATG
goat                                TTGATGAGCCCATAAAACAGAAAATGAACCACCTGGTGAATACTAAGGTTGACAAAGAGGACAACTTCTCTCAGCAAAATGCCAGTACGTTCCACCTCCTTGCTCAGAATCTGAGATATG
sheep                               TTGATGAGCCCATAAAACAGAAAATGAATCACTTGGTGAATACTAAGGTTGACAAAGAGGACAACTTCTCTCAGCAAAATGCCAGTACATTCCACCTCCTTGCTCAGAATCTGAGATATG
mouflon                             TTGATGAGCCCATAAAACAGAAAATGAATCACTTGGTGAATACTAAGGTTGACAAAGAGGACAACTTCTCTCAGCAAAATGCCAGTACATTCCACCTCCTTGCTCAGAATCTGAGATATG


pig                                 ATGAAACCAATCAGAGTTGTCACTTCCAAGAGGGATTTGCTGATCCCATTCACGCTGAAGTAAGTGGCACAGATATGATGGACAACCTCCCAGACCTTATCACTCAATGTATTCAGGTGG
killer_whale                        ATGAAACCAATCAGAGTGATCACTTCCAAGAGGAACTAGCTGACTCCATTCAGGCTGAAGTAAATGGCACAGATCTGATGGACAATCTCCCAGACCTTATCACTCAGTGTATTCAGTTGG
bottlenosed_dolphin                 ATGAAACCAATCAGAGTGATCACTTCCAGGAGGAACTAGCTGACTCCATTCAGGCTGAAGTAAATGGCACAGATCTGATGGACAATCTCCCAGACCTTATCACTCAGTGTATTCAGTTGG
Yangtze_river_dolphin               ATGAAGCAATCAGAGTGATCACTTCCAAGAGGAACTAGCTGACTCCATTCAGGCTGAAGTAAGTGGCACAGATCTGATGGACAATCTCCCAGACCTTATCACTCAGTGTATTCAGTTGG
sperm_whale                         ATGAAACCAATCAGAGTGATCACTTCCAAGAGGAGCTAGCTGACTCCATTCAGGCTGAAGTAAGTGGCACAGATCTGATGGACAATCTCCCAGACCTTATCACTCAGTGTATTCAGTTGG
Balaenoptera_acutorostrata_scammoni ATGAAACCAATCAGAGTGATCACTTCCAAGAGGAACTAGCTGACTCCATTCAGGCTGAAGTAAGTGGCACAGATCTGATGGACAATCTCCCAGACCTTATCACTCAGTGTATTCAGTTGG
water_buffalo                       ATGGATTCAATCAGAGTGATCACTTCCGAGAAGACCTAGCTGACCCCACTCAGGATGAAGTAAGTGGTCCAGATCTGTTGGACAATCTCCCAGACCTTATCACTCAGTGTATTCAGTTGG
Bison_bison_bison                   ATGGATTCAATCAGAGTGATCACTTCCAAGAAGAACTAGCTGACCCCACTCAGGATGAAGTAAGTGGTCCAGATCTGTTGGACAATCTCCCAGACCTTATTACTCAGTGTATTCAGTTGG
cattle                              ATGGATTCAATCAGAGTGATCACTTCCAAGAAGAACTAGCTGACCCCACTCAGGATGAAGTAAGTGGTCCAGATCTGTTGGACAATCTCCCAGACCTTATCACTCAGTGTATTCAGTTGG
wild_yak                            ATGGATTCAATCAGAGTGATCACTTCCAAGAAGAACTAGCTGACCCCACTCAGGATGAAGTAAGTGGTCCAGATCTGTTGGACAATCTCCCAGACCTTATCACTCAGTGTATTCAGTTGG
chiru                               ATGGATTCAATCAGAGTGATCACTTCCAAGA---ACTAACTGACTCCACTCAGGATGAAGTAAGTGGTCCAGATCCATTGGACAATCTCCCAGACCTTATCACTCAGTGTATTCAGTTGG
goat                                ATGGATTCAATCAGAGTGATCACTTCCAAGAAGAACTAACTGACTCCACTCAGGATGAAGTAAGTGGTCCAGATCCATTGGACAATCTCCCAGACCTTATCACTCAGTGTATTCAGTTGG
sheep                               ATGGATTCAATCAGAGTGATCACTTCCAAGAAGAACTAACTGACTCCACTCAGGATGAAGTAAGTGGCCCAGATCCATTGGACAATCTCCCAGACCTTATCACTCAGTGTATTCAGTTGG
mouflon                             ATGGATTCAATCAGAGTGATCACGTCCAAGAAGAACTAACTGACTCCACTCAGGATGAAGTAAGTGGCCCAGATCCATTGGACAATCTCCCAGACCTTATCACTCAGTGTATTCAGTTGG


pig                                 ACAAGAAACGTAGTAACAGGCCAGAGCTCCTACAGTCAGAGGCCCAGATCCCAGTGTTGGCTTCCCTGATCCACCACCAAACCCTCTCCAGCACCACAGGTCCACCACCTAAGGAAGACC
killer_whale                        ATAAGCAACGTAGTGACAGGCCAGAGCTCCTGCAGCCAGAGGCCCAGATCCCAGTGTTGGCTTCCCTGA---------------TCTCCAACCCCACAGGTCCACCACCCAAGGAAGACC
bottlenosed_dolphin                 ATAAGCAACGTAGTGACAGGCCAGAGCTCCTGCAGCCAGAGGCCCAGATCCCAGTGTTGGCTTCCCTGA---------------TCTCCAACCCCACAGGTCCACCACCCAAGGAAGACC
Yangtze_river_dolphin               ATAAGCAACGTAGTGACAGGCCAGAGCTCCTGCAGCCAGAGGCCCAGATCCCAGTGTTGGCTTCCCTGATCCATCACCAAGCCCTCTCCAACCCGACAGGTCCACCACCCAAGGAAGACC
sperm_whale                         ATAAGCAACGTAGTGACAGGCCAGAGCTCCTGCAGTCAGAGGCCCAGATCCCAGTGTTGGCTTCCCTGATCCACCACCAAGCCCTCTCCAACCCCACAGGTCCACCACCCAAGGAAGACC
Balaenoptera_acutorostrata_scammoni ATAAGCAACGTAGTGACAGGCCAGAGCTCCTGCAGTCAGAGGCCCAGATCCCAGTGTTGGCTTCCCTGATCCGCCACCAAGCCCTCTCCAACCCCACAGGTCCACCACCCAAGGAAGACC
water_buffalo                       ACAAGAAACACAGTGACTGGCCAGAGCTTCTAGAGTCAGAGGTCCAGATGCCAGTGTTGGCTCGCTGGATCCACCACCAAGCCTTCTCCAACCCCACAGGTCCAGCACCCAAGGAAGACC
Bison_bison_bison                   ACAAGAAACACAGTGAGTGGCCAGAGCTTCTAGAGTCAGAGGTCCAGATGCCAGTGTTGGCTCGCTGGATCCACCACCAAGCCTTCTCCAACCCCACAGGTCCAGCACCCAAGGAAGACC
cattle                              ACAAGAAACACAGTGAGTGGCCAGAGCTTCTAGAGTCAGAGGTCCAGATGCCAGTGTTGGCTCGCTGGATCCACCACCAAGCCTTCTCCAACCCCACAGGTCCAGCACCCAAGGAAGACC
wild_yak                            ACAAGAAACACAGTGAGTGGCCAGAGCTTCTAGAGTCAGAGGTCCAGATGCCAGTGTTGGCTCGCTGGATCCACCACCAAGCCTTCTCCAACCCCACAGGTCCAGCACCCAAGGAAGACC
chiru                               ACAAGAAACATAGTGACTGGCCAGAGCTTCTAGAATCAGAGGTCCAGATGCCAGTGTTGGCTCACTGGATCCACCACCAAGCCTTCTCCAACCCCACAGGTACAGCACCCAAGGAAGACC
goat                                ACAAGAAACATAGTGACTGGCCAGAGCTTCTAGAGTCAGAGGTCCAGATGCCAGTGTTGGCTCGCTGGATCCACCACCAAGCTTTCTCCAACCCCACAGGTCCAGCACCCAAGGAAGACC
sheep                               ACAAGAAACATAGTGACTGGCCAGAGCTTCTAGAGTCAGAGGTCCAGATGCCAGTGTTGGCTCGCTGGATCCACCACCAAGCTTTCTCCAACCCCACAGGTCCAGCACCCAAGGAAGACC
mouflon                             ACAAGAAACATAGTGACTGGCCAGAGCTTCTAGAGTCAGAGGTCCAGATGCCAGTGTTGGCTCGCTGGATCCACCACCAAGCTTTCTCCAACCCCACAGGTCCAGCACCCAAGGAAGACC
```

Fig. S3 (continued)

```
pig                                   CTGTACAGCTGCGAGGAGGCCAGCCACCTCTCACTCCAGCCAAACGAGCTCGCCAGCAAGAAACACACTCTTGCCTCTACTGCAGCCAGTCTGGTCATTTCACAAAAGATTGCCTTGCCA
killer_whale                          CTATACAGCTTCGAGGAAGCCAGCTGCCTCTTACTCCAGCCAAACGAGCCCGCCAGCAAGAAACTCAGTCATGCCTTTACTGCAGCCAGGCTGGTCACTTCACAAGAGATTGCCTTGCCA
bottlenosed_dolphin                   CTATACAGCTTCGAGGAAGCCAGCTGCCTCTTACTCCAGCCAAACGAGCCCGCCAGCAAGAAACTCAGTCATGCCTCTACTGCAGCCAGGCCGGTCACTTCACAAGAGATTGCCTTGCCA
Yangtze_river_dolphin                 CTATACAGCTTCGAGGAAGCCAGCTGCCTCTCACTCCAGCCAAACGAGCCCGACAGCAAGAAACTCAGTCATGCCTCTACTGCAGCCAGGCTGGTCACTTCACAAGAGATTGCCTTGCCA
sperm_whale                           CTATACAGCTTCGAGGAAGCCAGCCGCCTCTCACTCCAGCCAAACGAGCCCGCCAGCAAGAAACTCAGTCATGCCTCTACTGCAGCCAGGCTGGTCACTTCACAAGAGATTGCCTTGCCA
Balaenoptera_acutorostrata_scammoni   CTATACAGCTTCGAGGAAGCCAGCCGCCTCTCACTCCAGCCAAACGAGCCCGCCAGCAAGAAACTCAGTCATGCCTCTACTGCAGCCAGGCTGGTCACTTCACAAGAGATTGCCTTGCCA
water_buffalo                         GTATACAGCTGCGAGGAAGCCAGCCACCTCTCACTCCAGCCAAGCGAGCCCGCCAGCAAGAAGTTCAGGCATGCCTCTACTGCAGCCAGGCTGGTCACCTCACATTAGATTGCCTTGCCA
Bison_bison_bison                     GTGTACAGCTTCGAGGAAGCCAGCCACCTCTCACTCCAGCCAAACGAGCCCGCCAGCAAGAAGTTCAGGCATGCCTCTACTGCAGCCAGGCTGGTCATGTCACATTAGATTGCCTTGCCA
cattle                                GTATACAGCTGCGAGGAAGCCAGCCACCTCTCACTCCAGCCAAACGAGCCCGCCAGCAAGAAGTTCAGGCATGCCTCTACTGCAGCCAGGCTGGTCATGTCACATTAGATTGCCTTGCCA
wild_yak                              GTATACAGCTGCGAGGAAGCCAGCCACCTCTCACTCCAGCCAAACGAGCCCGCCAGCAAGAAGTTCAGGCATGCCTCTACTGCAGCCAGGCTGGTCATGTCACATTAGATTGCCTTGCCA
chiru                                 CTATACAGCTGCGAGGAAGCCAGCCACCTCTCACTCCAGCCAAACGAGCCCACCAGCAAGAAGTTCAGGCATGCCTCTACTGCAGCCAGGCTGGTCACCTCACATTAGATTGCCTTGCCA
goat                                  CTATACAGCTGCGAGGAAGCCAGCCACCTCTCACTCCAGCCAAACGAGCCCACCAGCAAGAAGTTCAGGCATGCCTCTACTGCAGCCAGGCTGGTCACCTCACATTAGATTGCCTTGCCA
sheep                                 CTATACAGCTGCGAGGAAACCAGCCACCTCTCACTCCAGCCAAACGAGCCCACCAGCAAGAAGTTCAGGCATGCCTCTACTGCAGCCAGCCTGGTCACCTCACATTAGATTGCCTTGCCA
mouflon                               CTATACAGCTGCGAGGAAACCAGCCACCTCTCACTCCAGCCAAACGAGCCCACCAGCAAGAAGTTCAGGCATGCCTCTACTGCAGCCAGCCTGGTCACCTCACATTAGATTGCCTTGCCA


pig                                   AACGTTCTCGAGCCCCAACAAGGACAAATAACTCATCTCAACAGTAA
killer_whale                          AACGTTCTCGAGCCCCAGCAAGGATAAATAACCCAGCTCACCAGTAA
bottlenosed_dolphin                   AACGTTCTCGAGCCCCAGCAAGGATAAATAACCCAGCTCACCAGTAA
Yangtze_river_dolphin                 AACGTTCTCGAGCCCCAGCAAGGATAAATAACCCAGCTCACCAGTAA
sperm_whale                           AACGTTCTCGAGCCCCAGCAAGGATAAATAACCCAGCTCACCAGTAA
Balaenoptera_acutorostrata_scammoni   AACGTTCTCGAGCCCCAGCAAGGATAAATAACCCAGCTCACCAGTAA
water_buffalo                         AACGTTCTCGAGCCCCAGCAAGGATAAATAACCCAACTCATCAGTAA
Bison_bison_bison                     AACGTTCTCGAGCCCCAGAAAGGATAAATAACCCAACTCATCAGTAA
cattle                                AACGTTCTCGAGCCCCAGAAAGGATAAATAACCCAACTCATCAGTAA
wild_yak                              AACGTTCTCGAGCCCCAGAAAGGATAAATAACCCAACTCATCAGTAA
chiru                                 AACGTTCTCGAGCCCCAGCAAGGATAAATAACCCAACTCATCAGTAA
goat                                  AACGTTCTCGAGCCCCAGCAAGGATAAATAACCCAACTCATCAGTAA
sheep                                 AACGTTCTCGAGCCCCAGCAAGGATAAATAACCCAACTCATCAGTAA
mouflon                               AACGTTCTCGAGCCCCAGCAAGGATAAATAACCCAACTCATCAGTAA
```

Fig. S3 (continued)

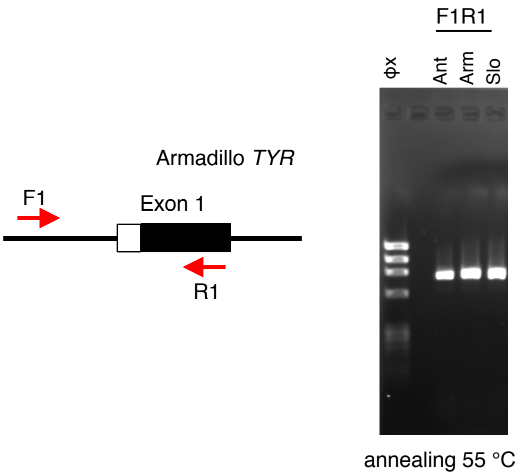

Fig. S4

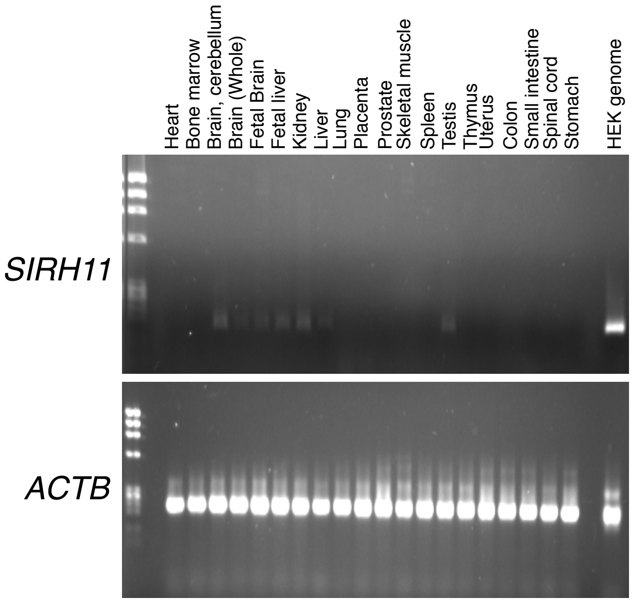
Heart
Bone marrow
Brain, cerebellum
Brain (Whole)
Fetal Brain
Fetal liver
Kidney
Liver
Lung
Placenta
Prostate
Skeletal muscle
Spleen
Testis
Thymus
Uterus
Colon
Small intestine
Spinal cord
Stomach
HEK genome
SIRH11
ACTB

Fig. S5
